# Molecular states and interfacial interactions at solid–liquid interfaces: advances and challenges in multimodal characterization

**DOI:** 10.3762/bjnano.17.85

**Published:** 2026-09-03

**Authors:** Shiou Yamada, Hinata Urano, Koji Toma

**Affiliations:** 1 Graduate School of Engineering and Science, Shibaura Institute of Technology, 3-7-5 Toyosu, Koto-ku, Tokyo 135-8548, Japan; 2 College of Engineering, Shibaura Institute of Technology, 3-7-5 Toyosu, Koto-ku, Tokyo 135-8548, Japan

**Keywords:** biointerfaces, molecular interactions, multimodal characterization, solid–liquid interfaces, spatiotemporal dynamics

## Abstract

Solid–liquid interfaces govern key functions in biosensing, catalysis, energy conversion, drug delivery, and organic electronic devices because molecular adsorption, hydration, charge regulation, and structural reorganization occur within an ultrathin interfacial region. However, these phenomena are intrinsically multiscale and multidimensional, involving strongly coupled variables such as mass, interfacial potential, molecular orientation, viscoelasticity, and local nanoscale heterogeneity. As a result, no single measurement technique can fully describe interfacial states or their dynamics. This review summarizes recent advances in multimodal characterization strategies designed to overcome these limitations. We first outline the conceptual framework of molecular interactions at solid–liquid interfaces, focusing on electrostatic interactions, van der Waals forces, hydration structures, hydrophobic effects, specific adsorption, and protein conformational changes across wide temporal and spatial scales. We then compare the observation windows, defined here as the combined range of spatiotemporal resolution and accessible physical observables, and limitations of major stand-alone methods, including optical, electrochemical, acoustic, and scanning probe techniques. Building on this basis, we discuss the recent development of multimodal platforms that integrate optics with electrochemistry, optics with quartz crystal microbalance measurements, and local probes with optical or electrochemical readouts, highlighting how these combinations reveal correlations among dry and wet mass, charge state, molecular vibration, hydration, and local structure. Finally, we discuss remaining challenges, including causal ambiguity among coupled interfacial variables, the trade-off between spatial and temporal resolution, and the difficulty of quantitatively linking experimental data with theoretical models. Multimodal characterization is expected to provide a foundation for the rational design of next-generation biointerfaces, delivery systems, and organic devices.

## Review

### 1 Introduction

Solid–liquid interfaces play a central role in a wide range of fields that underpin modern science and technology, including biosensors, catalysis, energy devices, and drug delivery systems. In the ultrathin region where an electrolyte solution contacts a solid surface, a unique molecular order forms that cannot be reduced to either the liquid phase or the solid phase alone, and this order essentially determines the function of the whole system. For example, in biosensors based on antigen–antibody reactions, orientation, density, and hydration state of surface-modifying molecules strongly affect signal sensitivity. In drug delivery using lipid nanoparticles, the dynamics of membrane fusion and cargo release at the solid–liquid interface determine carrier performance. In organic thin-film devices as well, charge accumulation and molecular conformational changes at the interface between the gate electrolyte and the semiconductor layer govern the field effect. In this way, a molecular-level understanding of solid–liquid interfaces provides a universal basis for the design of next-generation devices and materials.

The fundamental reason why molecular phenomena at solid–liquid interfaces are so complex is the diversity and interdependence of the interactions operating there. Electrostatic interactions, through the electrical double layer (EDL) formed cooperatively by surface charges and electrolyte ions, influence the orientation of adsorbed molecules and the ease of their desorption. van der Waals forces contribute to the early stages of adsorption as weak attractions among molecules and between molecules and substrates, while hydrophobic interactions promote the association of nonpolar sites by driving out water molecules. In addition, hydration structures, that is, multilayer shells of oriented water molecules formed near solid surfaces, not only create a dehydration energy barrier that must be overcome for direct molecular contact, but their structural changes themselves are also deeply involved in molecular orientation and function. These interactions do not vary independently; rather, they are modulated cooperatively as interlinked state variables. For example, changes in solution pH or salt concentration alter not only the interfacial charge density, but also the reorganization of hydration shells around adsorbed proteins, their conformational changes, and the viscoelastic properties of the entire adsorbed layer. A useful starting point for understanding solid–liquid interfaces is therefore to regard them not as a simple boundary, but as a nonlinear feedback system in which multiple physical quantities are strongly coupled.

Another essential feature of interfacial phenomena is their hierarchical time and length scales. Among the fastest processes, formation of the EDL and reorientation of water molecules proceed on timescales from picoseconds to microseconds [1–2], followed by the initial adsorption of molecules involving dehydration on timescales from nanoseconds to minutes [3–4]. Conformational relaxation and reorientation after adsorption have long time constants ranging from tens of minutes to hours [5–6], while collective membrane formation and phase transitions occur on timescales from seconds to hours [7–8]. In terms of length scale, the hydration shell formed by a single water molecule at a surface extends from angstroms to several nanometers, adsorption structures of proteins are on the nanometer scale, and heterogeneous distributions of reactive sites can extend to the micrometer scale. Integrating interfacial phenomena across such broad temporal and spatial scales is precisely where any single measurement method reaches its limits.

To determine the state of an interface accurately, it is necessary to evaluate multiple physical quantities dynamically, including mass and adsorption amount, charge state and interfacial potential, molecular vibrations and chemical structure, viscoelasticity and membrane structure, and local morphology and nanostructure. Traditionally, each of these has been studied by a dedicated stand-alone technique. Surface plasmon resonance (SPR) can measure adsorbed mass and binding kinetics with high sensitivity through refractive index changes near the interface, but it does not provide chemical structure information [9–10]. Quartz crystal microbalance with dissipation monitoring (QCM-D) can evaluate the “wet mass”, including hydration water, together with viscoelasticity, but it cannot directly detect molecular orientation or local charge [11–12]. Infrared (IR) spectroscopy and vibrational sum-frequency generation (VSFG) spectroscopy provide information on the vibrations and orientation of interfacial molecules, but in principle only as ensemble-averaged responses [13–14]. Scanning probe microscopy (SPM) provides local nanoscale morphological and mechanical information, but real-time dynamic measurements are limited [15–17]. Electrochemical methods can follow changes in interfacial charge and potential with high time resolution, but offer poor spatial information [18–19].

Each stand-alone method has its own intrinsic “observation window”. Here, we define this term as the combined range of spatiotemporal resolution and the specific physical observables accessible to each technique. Accordingly, any single method provides only one cross section of the multidimensional state space of an interface. For example, the question “How much of the adsorbed mass comes from hydration water?” becomes quantifiable only when SPR and QCM-D are combined to analyze the difference between dry mass and wet mass [12,20]. Likewise, the question “How does the adsorption orientation of molecules at an electrode interface change dynamically with potential?” can be answered clearly only by integrating surface-enhanced Raman spectroscopy (SERS) with electrochemical measurements to track potential-dependent changes in specific vibrational modes [21–22]. Thus, the importance of multimodal characterization, that is, obtaining multiple physical quantities in a complementary manner from the same interface on the same time axis, follows directly from the intrinsically multidimensional nature of interfacial phenomena. Against this background, multimodal measurement systems that integrate methods based on different physical principles on the same interface have developed rapidly in recent years. The essential significance of multimodal integration is not simply that it increases the number of measurable quantities. Rather, it redefines interfacial phenomena, which were previously summarized only as single mass changes or current responses, in terms of the correlation structure among multiple physical quantities, thereby providing a clue to uncovering their underlying causal relationships.

This review systematically organizes recent progress in multimodal characterization of solid–liquid interfaces and discusses both the technical achievements and the remaining conceptual challenges. Section 2 outlines the conceptual framework for molecular states and interactions at solid–liquid interfaces and presents an overall picture of the physical quantities that should be measured. Section 3 surveys the principles and inherent limitations of existing stand-alone methods. Section 4 explains the major multimodal architectures in different categories, namely, optics combined with electrochemistry/acoustics and local probes integrated with optics/electrochemistry, and presents their spatiotemporal observation windows and representative applications. Section 5 then summarizes the essential limitations faced by current measurement technologies and discusses prospects for overcoming them.

### 2 Conceptual framework for molecular states and interactions at solid–liquid interfaces

#### 2.1 Major intermolecular interactions at solid–liquid interfaces

**Figure 1:**
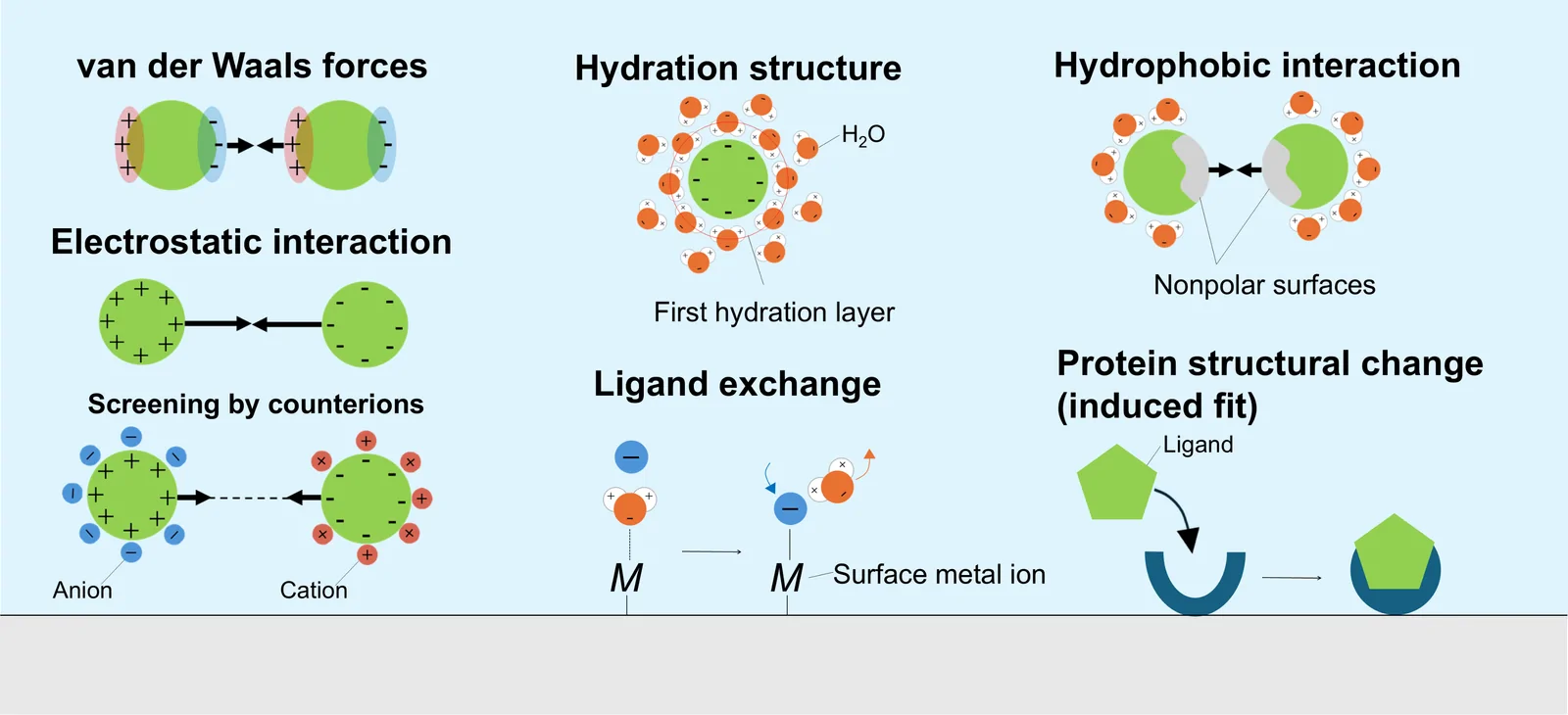
Summary schematic of intermolecular and interfacial interactions at solid–liquid interfaces.

**2.1.1 Electrostatic interactions.** Electrostatic interactions are forces that arise through space from the net charges or dipole moments carried by particles and molecules. They include charge–charge repulsion as well as charge–dipole and dipole–dipole interactions, which act over relatively short distances and can be attractive [23]. During molecular approach and binding, these forces dominate the early stage before the first hydration shells of the two partners come into contact, functioning either as the driving force for binding (attraction) or as an energy barrier (repulsion) [24]. In electrolyte solutions, counterions gather to neutralize the surface charge at the interface, forming an EDL composed of a Stern layer, where the finite size of ions causes steric exclusion, and an outer diffuse layer, where ions are distributed nonuniformly in space [25]. As a result, an interfacial potential distribution is established that decays from the charged interface into the solution. Owing to this screening by the EDL, the effective range over which surface charge exerts influence is limited (Figure 1), and this characteristic screening distance is defined as the Debye length [26]. When the ionic strength of the solution increases, screening by counterions becomes stronger and the Debye length becomes shorter, thereby changing both the range and the strength of electrostatic interactions [24]. In this way, electrostatic interactions determine the final balance between attraction and repulsion that governs whether binding can occur before shorter-range interactions come into play [24,26].

**2.1.2 Van der Waals forces.** Van der Waals forces are electromagnetic interactions that act between all atoms and molecules, and they consist of Keesom, Debye, and London dispersion forces (Figure 1). For a single pair of molecules, the interaction energy decays with the sixth power of distance. However, when macroscopic bodies are involved at solid–liquid interfaces, spatial integration changes the distance dependence. It decays with the third power of distance for a single molecule interacting with an infinite flat plate and is inversely proportional to distance in the near-contact region between spherical particles [27]. These theories of distance dependence, however, are based on the ideal assumption of perfectly smooth surfaces. At real solid–liquid interfaces, microscopic surface roughness alters the interaction. Indeed, studies using atomic force microscopy to measure forces between extremely smooth hafnium oxide surfaces have shown that the strong van der Waals attraction predicted to dominate at close separation is not observed. This was attributed to the fact that physical contact between microscopic asperities on the two surfaces generates a new repulsive force that cancels the attractive dispersion force [28].

**2.1.3 Hydration structure and hydrogen-bond network.** Water molecules are polar because of the electronegativity difference between oxygen and hydrogen, and each molecule has two proton-accepting and two proton-donating sites. As a result, water molecules in the solid form a continuous tetrahedral hydrogen-bond network with an average coordination number slightly above four. At solid–liquid interfaces, however, this network is modified through interactions with charged surfaces, producing hydration structures in which orientation and mobility of water are constrained [29] (Figure 1). This hydration shell around a molecular surface forms a dense first hydration layer that is more compact than bulk water [24], and the ordering of these water molecules decays exponentially from the surface into the bulk [29]. Because this dense first hydration layer exists, direct molecular contact requires a dehydration process in which water molecules are stripped away from the interface. Dehydration from hydrophilic or charged surfaces is thermodynamically highly unfavorable, so this compact hydration layer acts as a strong energy barrier to contact. In contrast, dehydration from hydrophobic surfaces is energetically favorable and contributes as an attractive force that brings molecules together, namely hydrophobic interaction [24].

**2.1.4 Hydrophobic interactions and ion specificity (Hofmeister series).** Hydrophobic interaction refers to the attractive phenomenon in which hydrophobic surfaces associate with each other to reduce their contact area with water, thereby minimizing the large energetic penalty that arises when water molecules cannot form hydrogen bonds around exposed nonpolar surfaces [30] (Figure 1). This attraction is now known to have a nanoscale decay length [30–31]. Tabor and co-workers directly measured the force between liquid droplets after excluding all known surface forces and demonstrated that the force itself has an extremely short decay length of about 0.3 nm, close to the correlation length of water molecules [30]. This microscopic attraction drives hydrophobic collapse and macroscopic aggregation. Whether such processes proceed, however, is strongly influenced by ions from the Hofmeister series. Zhang examined the effect of the Hofmeister series on the lower critical solution temperature (LCST) of the thermoresponsive polymer poly(*N*-isopropylacrylamide) and showed that kosmotropic ions strongly promote aggregation, lowering the LCST, through enhanced water polarization and increased interfacial tension. By contrast, chaotropic ions both increase interfacial tension and bind directly to the polymer; this direct binding causes a salting-in effect that partially offsets the salting-out effect arising from interfacial tension, thereby suppressing the decrease in LCST and slowing aggregation [32].

**2.1.5 Ligand exchange.** Specific adsorption of molecules and anions at solid–liquid interfaces can be described as a surface complexation reaction analogous to complex formation in homogeneous solution. Its main mechanism is ligand exchange, in which water molecules or hydroxy groups coordinated to surface functional groups, such as surface metal ions on metal oxides, silanol groups on silica, or carboxyl groups in natural organic matter, are replaced by ligands in solution, such as anions [33] (Figure 1). Adsorbed states are broadly divided into strong inner-sphere complexes, in which the adsorbate binds directly to the surface functional group, and outer-sphere complexes, in which water molecules remain between them. In particular, for inner-sphere complex formation, intrinsic chemical affinity, such as Lewis acid–base interactions, acts as the dominant driving force, beyond purely electrostatic factors [29,33].

**2.1.6 Protein structural changes.** Protein conformational changes are observed prominently in specific molecular recognition [34–36] and in adsorption onto nonbiological solid surfaces [37–38]. For structural changes associated with molecular recognition, two main models are commonly discussed, namely, induced fit and conformational selection. In induced fit, a flexible binding site is deformed by the approach of the ligand so that the two become complementary (Figure 1). In conformational selection, the ligand binds to the most suitable state among multiple dynamic conformations already populated by the protein, shifting the overall equilibrium toward the bound state [39]. Maus and co-workers used single-molecule Förster resonance energy transfer to analyze the binding mechanisms of competitive inhibitors to two structurally very similar enzymes, the NS2B-NS3 proteases of Zika virus and dengue virus. They showed that the Zika protease follows an induced-fit mechanism, whereas the dengue protease follows conformational selection [35]. Distinct structural changes also occur during adsorption onto solid surfaces. For example, Roach and co-workers used quartz crystal microbalance (QCM) and grazing-angle Fourier transform infrared spectroscopy to measure mass changes and secondary-structure changes during serum–protein adsorption on hydrophobic and hydrophilic model surfaces, demonstrating a clear correlation between adsorption-induced loss of α-helical structure and surface affinity [37].

#### 2.2 Time scales and length scales

**Figure 2:**
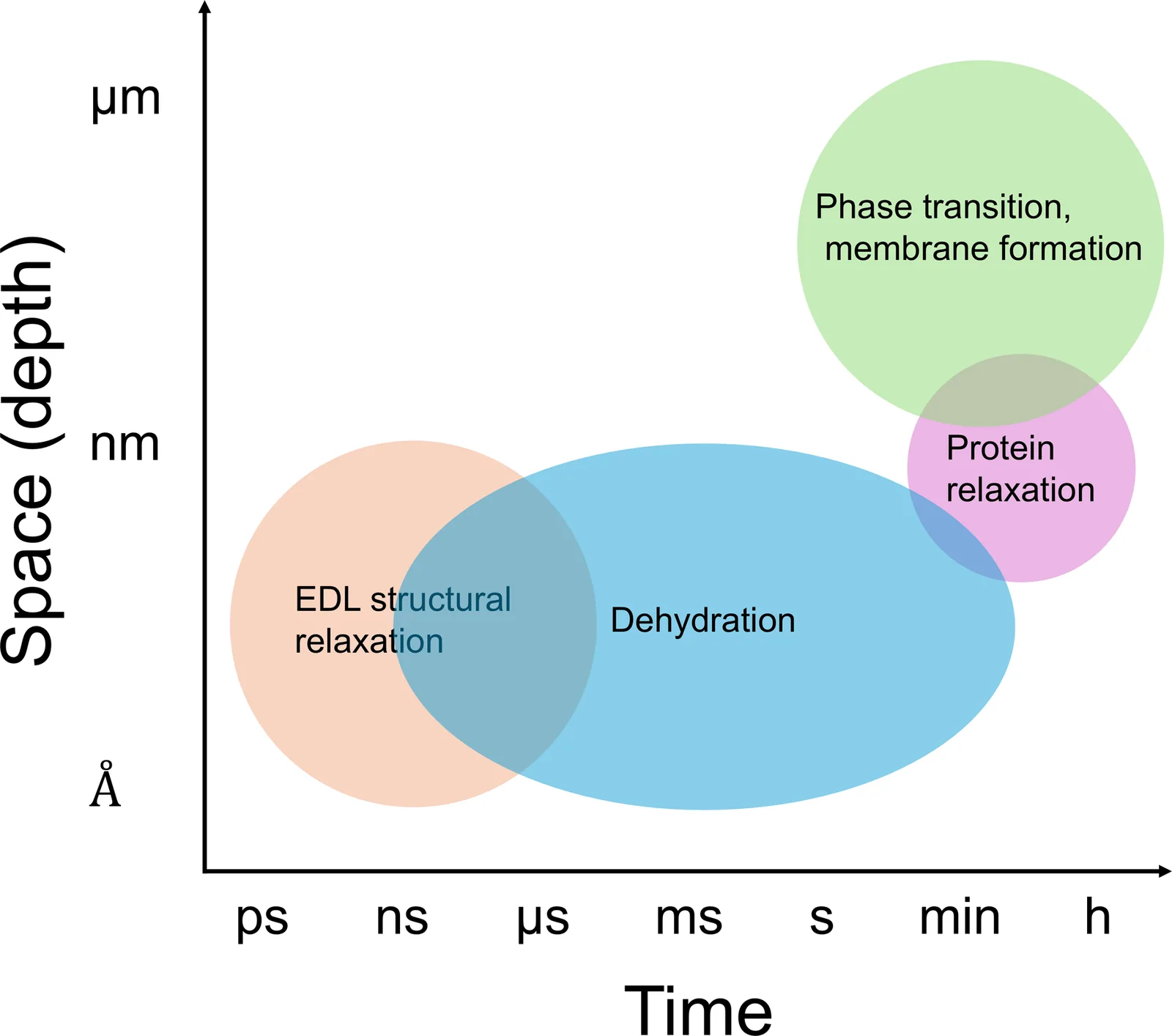
Spatiotemporal mapping of molecular and interfacial processes at solid–liquid interfaces.

**2.2.1 Structural relaxation of the electrical double layer (picoseconds to microseconds).** Structural relaxation of the EDL is the dynamic process by which an interface responds to a sudden environmental change, such as in electric field or temperature, and moves toward a new thermodynamic equilibrium over a spatial range from the angstrom-scale specific adsorption region to the diffuse layer several nanometers thick. Reported timescales span from picoseconds to microseconds (Figure 2) [1–2]. Yamakata and co-workers irradiated a CO-covered platinum electrode with a pulsed laser to thermally desorb CO and tracked the resulting relaxation of the EDL. They showed that adsorption of water molecules onto the newly exposed sites and their collective rearrangement required several tens of microseconds to relax into a new double-layer structure, and that the relaxation time became longer as the initial electrode potential decreased (about 20 μs at 0.4 V and about 70 μs at −0.3 V) [1]. In contrast, Greco and co-workers applied an ultrafast temperature-jump method using an infrared laser to the spontaneously formed EDL at the air–electrolyte solution interface, thereby isolating the intrinsic relaxation process of the double layer without interference from electrode materials. They demonstrated that structural relaxation of the EDL takes 20–200 ps depending on concentration and temperature and argued that this provides a benchmark for the response speed of solid-electrode devices [2].

**2.2.2 Protein structural relaxation (minutes to hours).** Protein structural relaxation at a solid–liquid interface is the process by which, after initial adsorption, a molecule changes its own conformation and orientation so as to maximize interactions with the surface and minimize the free energy of the system, thereby stabilizing it [37]. Model studies of proteins have reported that, for single molecules on the nanometer scale, the molecular footprint can expand over tens to hundreds of square nanometers on very slow timescales of tens of minutes to hours (Figure 2) [5–6]. Wertz and co-workers used total internal reflection fluorescence microscopy (TIRFM) to measure the amount of model proteins (albumin and fibrinogen) adsorbed on uncharged hydrophilic and hydrophobic surfaces; from these data, they calculated the time-dependent increase in the area occupied per molecule (the footprint). They found that the footprint of adsorbed molecules continued to increase at a nearly constant rate for at least the first 15–20 min, and that the relaxation process began to converge only after about 2 h. The rate and final extent of expansion depended strongly on surface hydrophobicity. Specifically, the increase was modest on hydrophilic surfaces, whereas on hydrophobic surfaces it was much larger, accompanied by unfolding of the internal structure, and the footprint expanded to three to five times its initial size [6].

**2.2.3 Collective phase transitions and membrane formation (seconds to hours).** Collective phase transitions and membrane formation at solid–liquid interfaces refer to processes in which molecules that are initially adsorbed in a random manner self-organize through intermolecular and molecule–surface interactions into thermodynamically stable ordered structures. Spatially, these processes range from local molecular arrangements and domain formation on the nanometer scale to macroscopic regions extending over several hundred micrometers, while temporally they span from seconds to hours depending on the system and concentration (Figure 2) [7–8,40–41]. Gobbi and co-workers used a graphene field-effect transistor (FET) to electrically track in real time the self-assembly dynamics of photoresponsive molecules at a solid–liquid interface. They showed that the rate of self-assembly of metastable merocyanine molecules generated by light irradiation depended strongly on molecular concentration near the interface, and that at a sufficiently high concentration (4 mM), membrane formation over a macroscopic area of 100 μm × 100 μm was completed rapidly on a timescale of seconds (time constant of 6 s) [40]. Son and co-workers also used scanning tunneling microscopy (STM) and X-ray photoelectron spectroscopy to observe the formation of self-assembled monolayers (SAMs) of amide-containing alkanethiols on Au(111). They visualized a transition from a structurally disordered liquid-like phase at the early stage of adsorption (1 min), to a mixed state after 10 min containing both ordered row structures (the α phase) and densely packed structures (the β phase), and finally to a uniform dense β phase after 1 h. They argued that this local structural transition is driven not only by increased sulfur chemisorption but also by rearrangement of the molecular backbone to maximize lateral intermolecular interactions [7].

**2.2.4 Dehydration during molecular approach to and adsorption at an interface (nanoseconds to minutes).** Molecules and ions in solution are thermodynamically stabilized by hydration shells, but in adsorption and related interactions, this hydration layer itself acts as a steric barrier that prevents direct contact. Therefore, for molecules and solid surfaces to approach each other and make direct contact, the hydration shell must be disrupted through a dehydration process [3]. Spatially, this dehydration is an interfacial process occurring over a highly localized region from angstroms to about 1 nm, and reported timescales range from nanoseconds to minutes (Figure 2) [3–4,42]. Camacho and co-workers used Brownian dynamics simulations to analyze binding between proteins for which long-range electrostatic attraction is weak. They found that within the dehydration layer, the average dehydration force is repulsive, limiting the lifetime of nonspecific collision complexes to about 4 ns. This suggests that under conditions without strong electrostatic steering, the search process involving molecular contact and dehydration takes place within a very short nanosecond time window [42]. By contrast, adsorption onto a solid–liquid interface under an external driving force can require much longer times. Yamakata and co-workers combined an electrochemical system with surface-enhanced infrared absorption spectroscopy (SEIRAS) to follow the approach of cations with hydrophobic hydration shells to a CO-covered platinum electrode. They showed that the rate of hydration-shell breakdown depends on the applied driving force (potential) and that even under strong driving conditions, the breakdown of the hydration shell required about 185 ms [3]. Maitra and co-workers further monitored the approach and adsorption of multivalent ions to probe molecules on a gold electrode by IR spectroscopy, demonstrating a delayed process that required about 11 min for dehydration in aqueous solution, and arguing that dehydration itself can become the rate-limiting step in adsorption [4].

#### 2.3 Physical quantities that should be measured

**2.3.1 Mass.** Mass is a fundamental physical quantity for evaluating adsorption and desorption at solid–liquid interfaces. Real-time tracking of its temporal changes enables the evaluation of the kinetics and binding affinity of intermolecular interactions [43]. Furthermore, measuring mass changes in response to varied surface properties or solution conditions confirms whether molecular adsorption occurs under specific conditions, providing a key indicator for identifying the driving forces behind interfacial interactions [44–45].

**2.3.2 Charge state and interfacial potential.** Charge states and interfacial potentials are physical quantities that reflect the electrostatic environment at solid–liquid interfaces. Capturing these variables allows for the evaluation of a series of physicochemical processes that alter this electrostatic environment, such as the adsorption of target molecules and ions, as well as the conformational changes of probe molecules [46–48].

**2.3.3 Molecular vibrations.** The intrinsic vibrational states of molecules serve as chemical fingerprints for molecular identification. Analyzing these vibrational states therefore enables the characterization of chemical species, molecular structures, and hydrogen-bonding networks at solid–liquid interfaces [49–50].

**2.3.4 Viscoelasticity.** The viscoelasticity of the adsorbed layer formed at a solid–liquid interface is a physical quantity that characterizes the mechanical flexibility and hydration state of the molecular layer. In practical measurements, it serves as an indicator for evaluating the structural changes of adsorbed proteins or the growth processes of polymer layers [51–52].

**2.3.5 Local structure (nanometer scale).** Local structure is a physical parameter that reflects the spatial heterogeneity of solid–liquid interfaces, details that are often obscured in macroscopic, averaged measurements. Capturing this local structure makes it possible to extract features such as island-like clusters, fibril networks, and localized defects within adsorbed layers that might otherwise be perceived as uniform films [53–56].

These considerations clarify which physical quantities must be tracked to describe solid–liquid interfacial states in a meaningful way. The following section therefore reviews the major stand-alone measurement methods, focusing on what each technique can reveal and where its intrinsic limitations remain.

### 3 Existing stand-alone measurement methods

#### 3.1 Optical Methods

**Figure 3:**
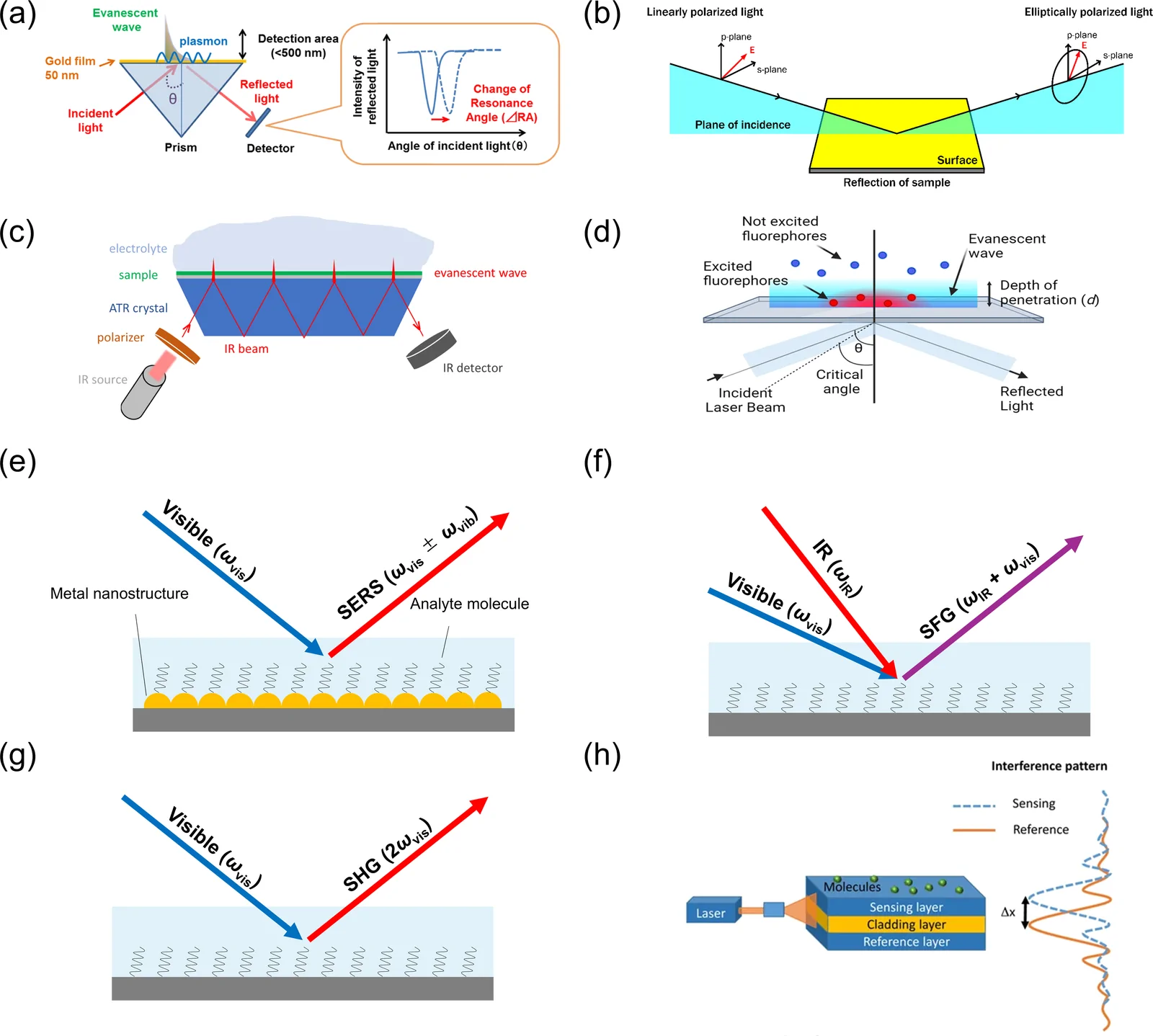
Schematic illustrations of measurement principles for optical methods. (a) SPR spectroscopy. Figure 3a was reproduced from [79] (“Surface Plasmon Resonance for Cell-Based Clinical Diagnosis”, © 2014 Y. Yanase et al., published by MDPI, distributed under the terms of the Creative Commons Attribution 3.0 Unported License, https://creativecommons.org/licenses/by/3.0/). (b) Ellipsometry. Figure 3b was reproduced from [80] (© 2022 I. Plikusiene et al., published by MDPI, distributed under the terms of the Creative Commons Attribution 4.0 International License, https://creativecommons.org/licenses/by/4.0/). (c) ATR-IR spectroscopy. Figure 3c was reproduced from [81] (© 2021 A. Bieberle-Hütter et al., published by IOP Publishing, distributed under the terms of the Creative Commons Attribution 4.0 International License, https://creativecommons.org/licenses/by/4.0/). (d) TIRFM. Figure 3d was adapted from [82] (© 2023 L. Colson et al., published by MDPI, distributed under the terms of the Creative Commons Attribution 4.0 International License, https://creativecommons.org/licenses/by/4.0/). (e) SERS. (f) VSFG spectroscopy. (g) SHG spectroscopy. (h) DPI. Figure 3h was adapted from [76] with permission. Copyright 2014 American Chemical Society. This content is not subject to CC BY 4.0.

**3.1.1 Surface plasmon resonance spectroscopy.** Surface plasmon resonance (SPR) spectroscopy is a measurement method based on the resonant excitation of surface plasmons, which are collective oscillations of free electrons, when polarized light is incident on the interface between a metal (mainly gold or silver) and a dielectric medium (solution) (Figure 3a). The evanescent field generated at the metal surface is localized within about a few hundred nanometers of the interface and decays exponentially, making the method highly sensitive to local refractive-index changes near solid–liquid interfaces [10]. Using this high sensitivity, SPR can track changes in interfacial mass in a label-free and real-time manner and can therefore evaluate the binding dynamics of target molecules [9,57]. For example, Sarcina and co-workers used shifts in the SPR angle together with the de Feijter equation to calculate the surface coverage (mass per unit area) and evaluate the antibody modification process on SAMs on gold surfaces. They demonstrated that the antibody concentration required could be reduced to one tenth of the conventional level without loss of analytical performance, thereby optimizing the surface-modification protocol for biosensors [57].

**3.1.2 Ellipsometry.** Ellipsometry is a non-destructive method for evaluating thin-film thickness and optical constants by analyzing the change in polarization that occurs when obliquely incident linearly polarized light is reflected from a sample surface. Specifically, it measures the change in polarization upon reflection, which is described by the amplitude ratio (Ψ) and phase difference (Δ) between the p- and s-polarized components of the reflected light [58] (Figure 3b). By fitting an appropriate optical model to the experimental data, the thickness and refractive index of the target layer can be derived [59–60]. This method can therefore be used to evaluate the mass and binding dynamics of adsorbed molecules. Mora and co-workers used spectroscopic ellipsometry to study the adsorption of an enzyme, ᴅ-amino acid oxidase, on carbon nanotubes. By performing dynamic adsorption experiments while varying pH and protein concentration, they calculated the surface mass and the initial adsorption rate, showing that the adsorption process was governed by a combination of hydrophobic and electrostatic interactions. Furthermore, by comparing the optical film thickness derived from ellipsometry with enzyme activity measured separately by biochemical assay, they reported that differences in pH during adsorption caused changes in enzyme orientation and conformation, which in turn produced differences in catalytic activity [59].

**3.1.3 Infrared spectroscopy.** Infrared (IR) spectroscopy is a method that uses the resonant absorption of specific infrared light associated with molecular vibrations (Figure 3c). The resulting infrared absorption spectrum directly reflects the vibrational states of molecules and is highly sensitive to their local chemical structures [13]. This makes the method useful not only for identifying chemical species at interfaces, but also for evaluating secondary-structure changes, orientation, and intermolecular interactions of adsorbed molecules [37,61–62]. Roach and co-workers used attenuated total reflection infrared (ATR-IR) spectroscopy to evaluate the adsorption of bovine serum albumin (BSA) and fibrinogen on silica nanoparticles of various sizes. By deconvoluting the amide-I band arising from peptide bonds, they quantified how surface hydrophilicity or hydrophobicity and nanoscale surface curvature affect the relative amounts of α-helical and other structural components in the proteins. They also evaluated differences in molecular orientation from the intensity ratio of the amide-I and amide-II bands, and the strength of hydrogen bonding between neighboring adsorbed proteins from specific component peaks [62].

**3.1.4 Total internal reflection fluorescence microscopy.** Total internal reflection fluorescence microscopy (TIRFM) is an imaging method that uses the evanescent field generated when an incident light undergoes total internal reflection at a solid–liquid interface. This field selectively excites fluorescent molecules within a few hundred nanometers layer on the solution side of the interface, and the resulting weak emission is captured with a highly sensitive camera [63] (Figure 3d). From the fluorescence intensity and two-dimensional coordinates of bright spots recorded by the camera, the method can evaluate the number of adsorbed molecules, single-molecule adsorption and desorption kinetics, and microfluidic dynamics near the interface [63–65]. Lyu and co-workers used TIRFM to evaluate the desorption kinetics of BSA from amine-modified glass surfaces. Through microscopic observation at the single-molecule level, they found that the desorption pattern of BSA changed clearly around 400 s after adsorption, revealing a complex aging process in which the initial weakly bound state gradually converted into a long-lived strongly bound state [63].

**3.1.5 Raman spectroscopy.** Raman spectroscopy is a method that uses inelastic scattering generated when monochromatic light interacts with molecules, and evaluates molecular vibrational energy levels from the energy difference between the incident and scattered light (the Raman shift). For solid–liquid interface measurements, SERS, which combines electromagnetic enhancement due to localized surface plasmon resonance (LSPR) in metallic nanostructures with chemical enhancement arising from charge transfer between molecules and the substrate, is widely used [66] (Figure 3e). The Raman spectrum directly reflects the vibrational states of specific chemical bonds and is therefore sensitive to local chemical structure. As a result, this method can be used not only to identify target molecules but also to evaluate structural changes, orientation, and intermolecular interactions [67–69]. Brulé and co-workers used dynamic SERS and principal component analysis to evaluate conformational changes of single BSA molecules adsorbed on gold nanoparticles. By analyzing time-dependent fluctuations in Raman bands arising from hydrophobic amino acid residues such as tryptophan and tyrosine, they identified the dynamics by which BSA changes from a naturally folded state under physisorption to an unfolded chemisorbed state in which internal amino acids become exposed through hydrophobic interaction with the surface [67].

**3.1.6 Vibrational sum frequency generation spectroscopy.** Vibrational sum frequency generation (VSFG) spectroscopy is a nonlinear optical method in which visible and tunable infrared light are simultaneously irradiated onto an interface, and the emitted sum-frequency light is detected. Because signal generation is forbidden in centrosymmetric bulk liquids and amorphous media, only the signal from the symmetry-broken solid–liquid interface can be extracted without being buried in bulk noise [70] (Figure 3f). The resulting SFG spectrum (nonlinear susceptibility) directly reflects the vibrational states of interfacial molecules and is extremely sensitive to their microscopic local chemical structures. Accordingly, by analyzing vibrational spectra associated with specific functional groups at the interface, the method can evaluate not only chemical identity and hydrogen-bond networks but also changes in molecular structure and orientation, including absolute orientation [14,71–72]. Tian and co-workers used phase-sensitive VSFG spectroscopy to evaluate the hydrogen-bond network of water molecules at the interface between water and hydrophobic octadecyltrichlorosilane. By directly measuring the imaginary part of the interfacial vibrational spectrum while changing the pH of the bulk solution, they determined the absolute orientation of water molecules and demonstrated that hydroxide ions are specifically adsorbed at the interface even in nearly neutral pure water. They further clarified the dynamics by which the negative surface charge generated by these ions reorganizes the orientation of the hydrogen-bond network of interfacial water [71].

**3.1.7 Second harmonic generation (SHG) spectroscopy.** Second harmonic generation (SHG) spectroscopy is a nonlinear optical method in which light at twice the frequency of an incident single-wavelength beam is generated and detected at an interface (Figure 3g). As in SFG, the signal is generated only at interfaces where inversion symmetry is broken, giving SHG high interfacial selectivity [73]. The SHG signal directly reflects the electronic state of the interface and is highly sensitive to the interfacial electrostatic field. By tracking changes in SHG signal, macroscopic charging behavior associated with pH changes and acid–base equilibrium processes of interfacial molecules can be evaluated quantitatively [74–75]. Konek and co-workers studied the acid–base equilibrium of surface molecules at a carboxylic acid-modified silica/water interface. By analyzing titration curves of the field-induced SHG signal obtained while varying pH, they identified two deprotonation processes associated with different hydrogen-bonding states and quantified the acid dissociation constants for each [74].

**3.1.8 Dual-polarization interferometry.** Dual-polarization interferometry (DPI) is an optical technique that tracks nanoscale structural changes perpendicular to the solid–liquid interface in real time. It achieves this by independently measuring the phase shifts of transverse electric and transverse magnetic propagation modes, which are alternately introduced to the surface of a slab optical waveguide, as they respond to molecular adsorption and conformational changes at the interface (Figure 3h) [76]. The measured phase-shift data are converted into the thickness and refractive index of the adsorbed layer using an electromagnetic analytical model. This allows for the evaluation of the surface mass density and film density of the adsorbed molecules themselves, excluding the effects of hydration, as well as structural changes associated with protein adsorption and denaturation [77–78]. Zheng and co-workers exploited the ultrasensitive phase detection capability of DPI to evaluate the interaction between complement protein C1q, which is involved in the progression of Alzheimer’s disease, and the pathogenic molecule amyloid β42 (Aβ42). Specifically, they simultaneously and continuously tracked changes in mass, thickness, and density during the binding processes of C1q with different aggregation states of Aβ42. Their results revealed that Aβ42 monomers exhibit a particularly high affinity for C1q compared to other aggregated forms. Furthermore, during the binding of Aβ42 monomers, they observed a rapid initial adsorption followed by a gradual structural reorganization that formed a denser layer [78].

#### 3.2 Electrochemical Methods

**Figure 4:**
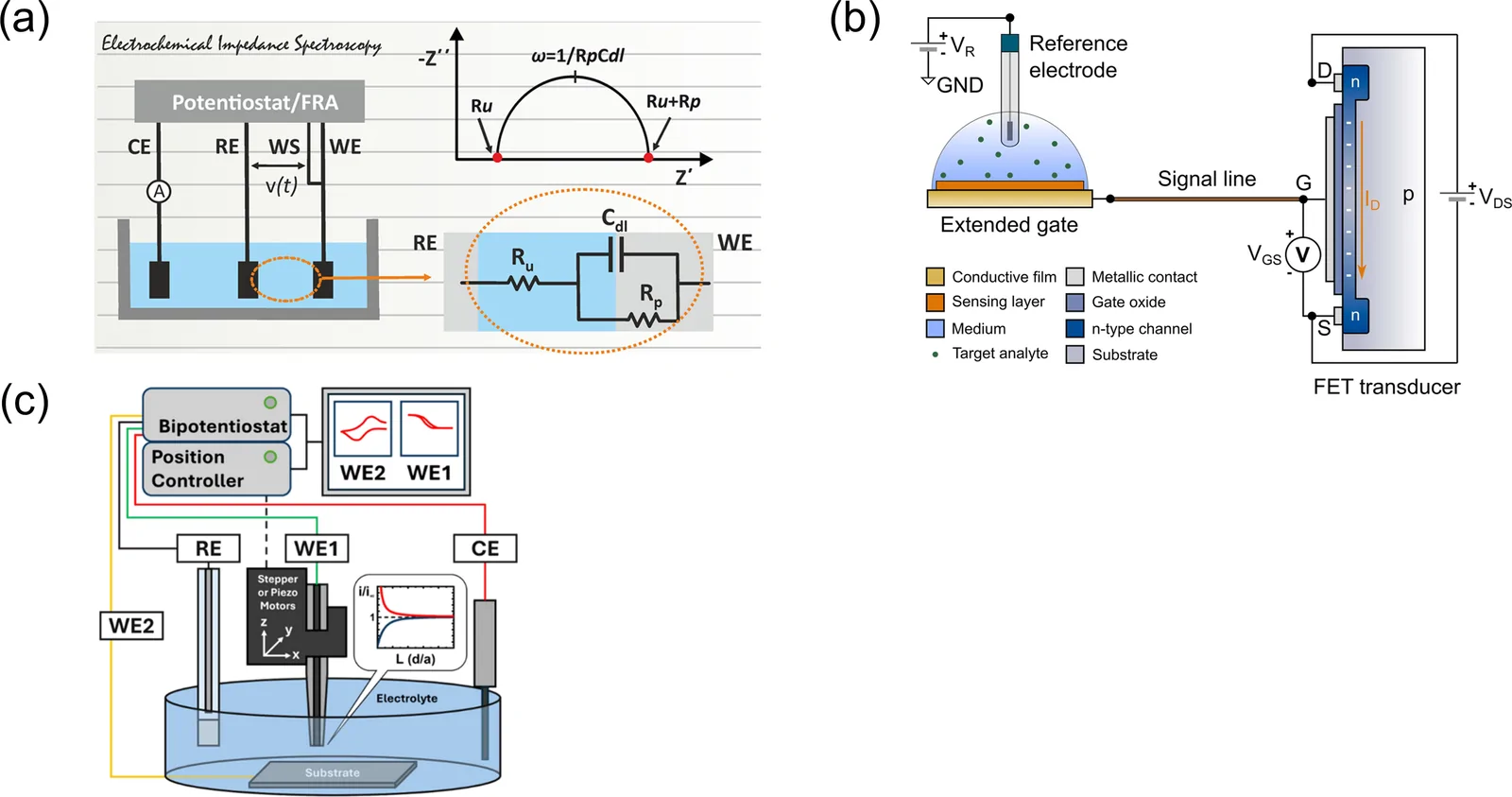
Schematic illustrations of measurement principles for electrochemical methods. (a) EIS. Figure 4a was reproduced from [83] (© 2023 A. Ch. Lazanas et al., published by American Chemical Society, distributed under the terms of the Creative Commons Attribution-NonCommercial-NoDerivatives 4.0 International License, http://creativecommons.org/licenses/by-nc-nd/4.0/). This content is not subject to CC BY 4.0. (b) ExG-FET. Figure 4b was reproduced from [90] (© 2023 Ž. Janićijević et al., published by Elsevier, distributed under the terms of the Creative Commons Attribution 4.0 International License, https://creativecommons.org/licenses/by/4.0/). (c) SECM. Figure 4c was adapted from [91] with permission. Copyright 2025 American Chemical Society. This content is not subject to CC BY 4.0.

**3.2.1 Electrochemical impedance spectroscopy.** Electrochemical impedance spectroscopy (EIS) is a method for evaluating the electrical properties of an interface by applying a small sinusoidal potential perturbation over a wide frequency range to an electrochemical system at steady state and measuring the impedance from the response current [83] (Figure 4a). In EIS, equivalent circuit parameters can be extracted by fitting the impedance response to an equivalent circuit model [83–84]. Magar and co-workers reported a point-of-care method for quantifying trace calcium ions in human saliva using a SAM-modified gold electrode. By analyzing EIS data acquired in the absence of a redox probe with a Randles equivalent circuit, they captured the increase in calcium concentration as a decrease in charge-transfer resistance and an increase in EDL capacitance. They further interpreted this response theoretically as a structural change in the EDL caused by specific ion adsorption, and finally derived the target concentration from the magnitude of the resistance decrease [84].

**3.2.2 Field-effect transistor.** A field-effect transistor (FET) converts changes in charge state and potential within the EDL at a solid–liquid interface directly into electrical signals, such as changes in semiconductor-channel conductance, through electrostatic interactions [85] (Figure 4b). This method can detect changes in interfacial charge state and potential caused by adsorption and desorption of target molecules or by charge rearrangement accompanying structural changes [18,86–87]. Nakatsuka and co-workers modified the surface of an ultrathin indium oxide FET with DNA aptamers that undergo adaptive structural changes upon target binding and achieved highly sensitive detection of small molecules under physiological conditions [18].

**3.2.3 Scanning electrochemical microscopy.** Scanning electrochemical microscopy (SECM) is a method in which a small probe (an ultramicroelectrode, UME) is scanned in solution close to the sample surface, and the Faradaic current associated with redox reactions between the probe and the substrate is measured [88] (Figure 4c). The resulting current response is influenced by local electrochemical properties at the sample surface, such as enzymatic activity and electronic conductivity, as well as by the surface charge state. Analysis of this current response therefore allows one to evaluate local electron-transfer processes and electrostatic interactions at the interface, while also mapping the spatial distribution of electrochemical properties [41,89]. Boldt and co-workers used SECM to measure local electron-transfer processes on a carboxyl-terminated SAM on a gold electrode. They showed that changes in the protonation and deprotonation state of the SAM surface with solution pH generate electrostatic interactions (Coulomb forces) with redox mediators carrying different charges, thereby modulating the electron-transfer rate at the interface [41].

#### 3.3 Mechanical response

**Figure 5:**
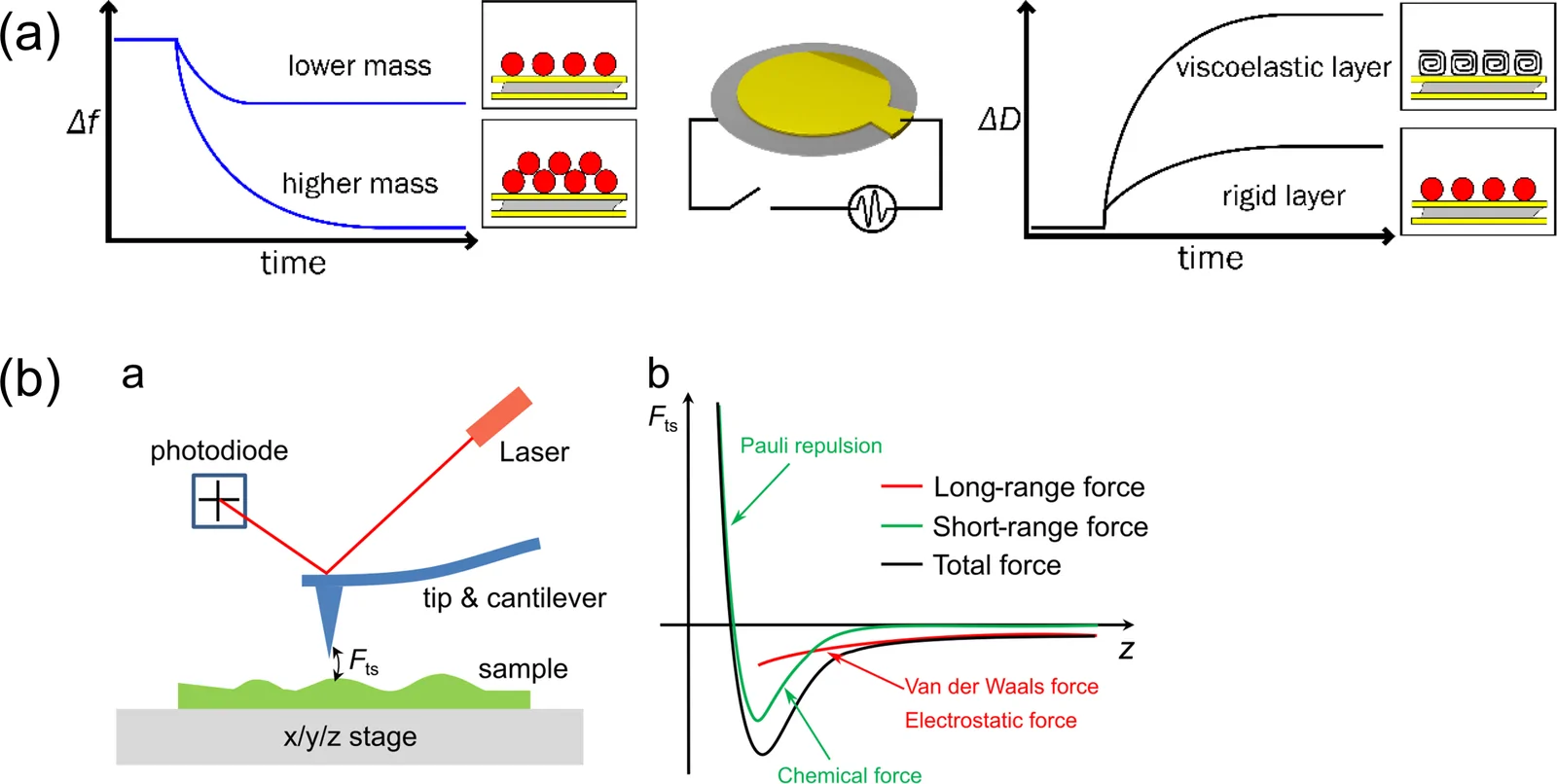
Schematic illustrations of measurement principles for mechanical methods. (a) QCM-D. Figure 5a was reproduced from [80] (© 2022 I. Plikusiene et al., published by MDPI, distributed under the terms of the Creative Commons Attribution 4.0 International License, https://creativecommons.org/licenses/by/4.0/). (b) AFM-based force spectroscopy. Figure 5b was reproduced from [15] (© 2021 J. Peng et al., published by Elsevier, distributed under the terms of the Creative Commons Attribution-NonCommercial-NoDerivatives 4.0 International License, https://creativecommons.org/licenses/by-nc-nd/4.0/). This content is not subject to CC BY 4.0.

**3.3.1 Quartz crystal microbalance with dissipation monitoring.** QCM-D is a label-free acoustic method that simultaneously measures the change in resonance frequency of a piezoelectric quartz crystal resonator and the energy dissipation of its vibration, thereby evaluating changes in surface mass, including hydration water, and the viscoelasticity of the system [11] (Figure 5a). From these measurements of hydrated mass and viscoelasticity, one can evaluate the binding dynamics, hydration state, and structural changes of molecules at interfaces [12,51,92]. For example, Lubarsky and co-workers used QCM-D to follow the hydration and dehydration process of a human serum albumin film adsorbed on a hydrophilic surface. Under dry conditions the adsorbed film showed low energy dissipation and behaved rigidly, whereas increasing ambient humidity promoted hydration, increased energy dissipation, and plastically transformed the film into a highly hydrated, flexible viscoelastic body. By applying the Voigt–Voinova model to the data, they further quantified the shear modulus and viscosity of the film, demonstrating that as the protein film absorbed water and gained mass through hydration, the entire adsorbed layer dynamically changed into a more elastic and flexible structure [92].

**3.3.2 Atomic force microscopy-based force spectroscopy.** Atomic force microscopy (AFM)-based force spectroscopy is a method in which a probe located at the tip of a flexible cantilever is moved only in the direction normal to the sample surface, and the attractive and repulsive forces between the probe and the surface are measured with piconewton sensitivity [11] (Figure 5b). By analyzing the force curves obtained during approach, indentation, and retraction, this method can quantitatively examine local mechanical properties (such as elastic modulus) and interaction forces at solid–liquid interfaces [93–94]. Andriotis and co-workers applied AFM nanoindentation in aqueous solution to collagen fibrils glycated by ribose treatment. By analyzing the unloading portion of the force curve with a continuum-mechanics model, they calculated the local elastic modulus (indentation modulus) of the sample. They showed on the nanoscale that the glycated collagen fibrils exhibited enhanced swelling due to hydration (water adsorption), and that the resulting decrease in molecular packing density significantly lowered the local elastic modulus compared with untreated collagen fibrils [94].

#### 3.4 Scanning probe microscopy

**Figure 6:**
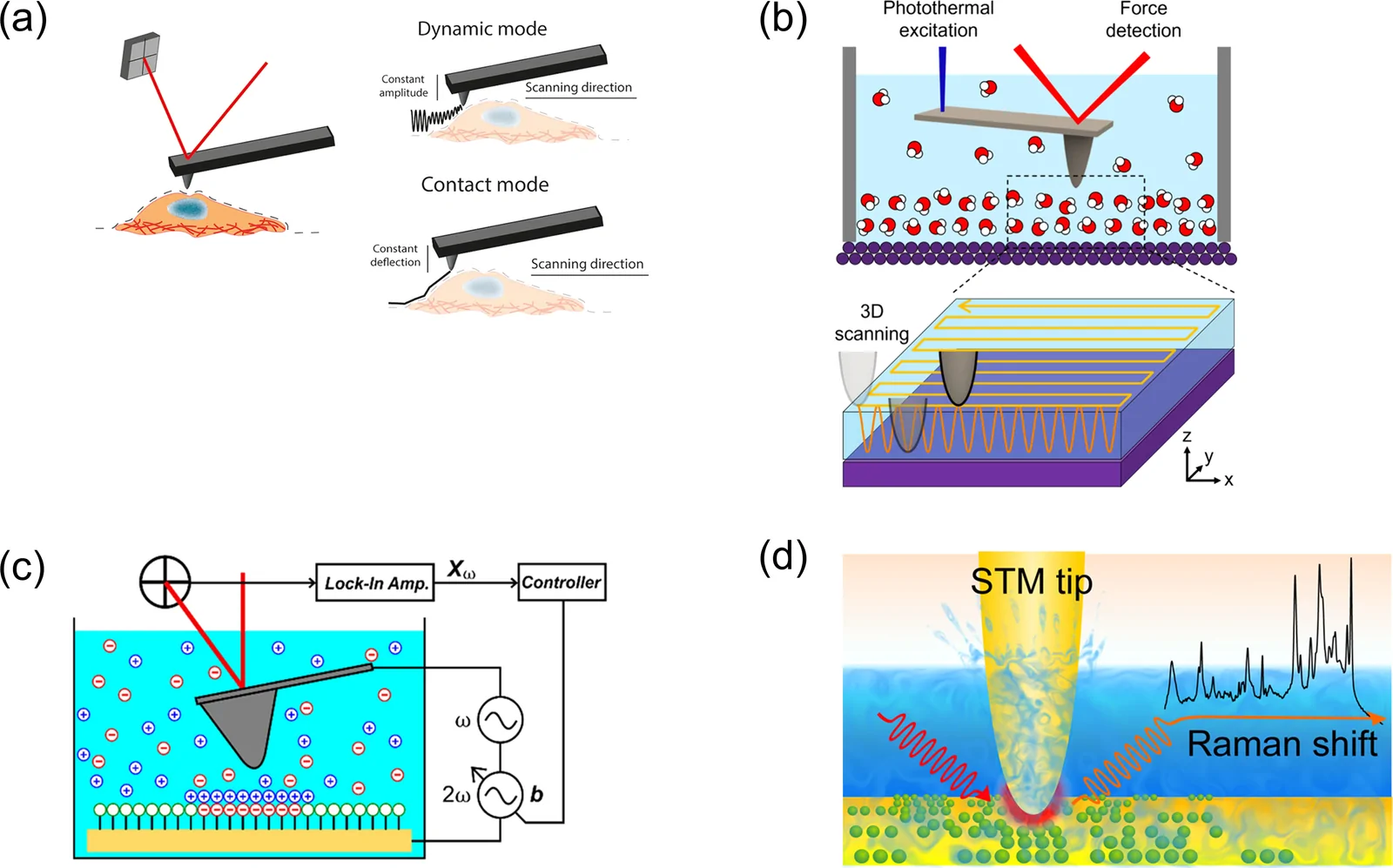
Schematic illustrations of measurement principles for SPM methods. (a) AFM. Figure 6a was adapted from [106] with permission. Copyright 2020 American Chemical Society. This content is not subject to CC BY 4.0. (b) 3D-AFM. Figure 6b was adapted from [107] with permission. Copyright 2025 American Chemical Society. This content is not subject to CC BY 4.0. (c) AC-KPFM. Figure 6c was adapted from [101] (© 2022 T. Hackl et al., published by American Chemical Society, distributed under the terms of the Creative Commons Attribution 4.0 International License, https://creativecommons.org/licenses/by/4.0/). (d) TERS. Figure 6d was reprinted from [108], N. M. Sabanés et al., “Versatile Side-Illumination Geometry for Tip-Enhanced Raman Spectroscopy at Solid/Liquid Interfaces”, Analytical Chemistry, © 2016 American Chemical Society, distributed under the ACS AuthorChoice/Editors’ Choice via Creative Commons CC-BY Usage Agreement, https://pubs.acs.org/page/policy/authorchoice_ccby_termsofuse.html. This content is not subject to CC BY 4.0.

**3.4.1 Atomic force microscopy.** AFM is a method that scans the sample surface while using feedback control based on the minute forces acting between the probe and the sample, thereby producing high-resolution images of local three-dimensional topography [11] (Figure 6a). On this basis, the method can evaluate local three-dimensional structures and their dynamic changes [95–96]. Nishide and co-workers used high-speed AFM, which greatly increases scanning speed, to visualize in real time the condensation of DNA mediated by protamine. They captured the dynamic process in which the addition of protamine first caused DNA to form local coil-like structures and then to fold stepwise into rod-like intermediates, leading them to propose a condensation model that culminates in dense toroidal structures. By tracking the response of the structure while increasing salt concentration, for example with NaCl, they also observed that the formed toroidal condensates and related structures disassembled in a stepwise and reversible manner, demonstrating that these dynamic structural changes are driven by electrostatic intermolecular interactions [95].

**3.4.2 Frequency-modulation AFM and three-dimensional AFM.** Frequency-modulation AFM (FM-AFM) is a method that evaluates local structures at solid–liquid interfaces by detecting the resonant frequency shift of a cantilever caused by the minute interaction forces acting between the probe and the sample in a liquid environment. Three-dimensional AFM (3D-AFM), which extends this technique to three-dimensional scanning, acquires the 3D spatial distribution of the resonant frequency shift and the interaction forces at the interface by scanning the *XY* plane while simultaneously rapidly modulating the probe in the *Z* direction [97] (Figure 6b). Based on these principles, both methods go beyond determining solid surface topography; they can evaluate hydration structures at the interface, the average conformation of lipid bilayer headgroups, and the molecular arrangement of SAMs with high spatial resolution [98–100]. Araki and co-workers used FM-AFM to observe the surface and hydration structures of a bioinert mixed-charged SAM (MC-SAM) in liquid at the molecular scale. They discovered that the molecules constituting the film form a highly ordered rectangular lattice array. Furthermore, they revealed the presence of a mechanically highly stable hydration layer near the MC-SAM surface, demonstrating that this robust hydration shell functions as a physical barrier preventing the adsorption of proteins and cells, thereby serving as the origin of its bioinert properties [100].

**3.4.3 Kelvin probe force microscopy.** Kelvin probe force microscopy (KPFM) is a method that detects the electrostatic force or its gradient acting between a conductive probe and a sample surface and determines the contact potential difference from the compensating voltage [16] (Figure 6c). Using this principle, the method can map the local surface-potential distribution at interfaces with high spatial resolution [101–102]. Hackl and co-workers used AC-KPFM, which employs a high-frequency AC voltage, to evaluate micropatterned SAMs in aqueous solution. They mapped the local surface-potential distributions of regions modified with amino and carboxyl groups to mimic biomolecules such as proteins. By changing solution pH, they controlled the ionization state of these functional groups and successfully measured the accompanying changes in charge state reversibly. Furthermore, from the polarity inversion and distance dependence of the measured local potential distribution, they inferred that counterions adsorb to the surface charge and form a Stern layer in aqueous solution, thereby revealing the spatial screening effect of the EDL that is characteristic of solid–liquid interfaces [101].

**3.4.4 Tip-enhanced Raman spectroscopy.** Tip-enhanced Raman spectroscopy (TERS) is a method that uses the intense electromagnetic near field enhanced by LSPR excited at the tip of a metallic SPM probe to obtain vibrational spectra of molecules with nanoscale spatial resolution [17] (Figure 6d). By analyzing these spectra, the method can evaluate local chemical composition, spatial heterogeneity, chemical reaction processes, and structural changes and orientation [103–105]. El-Khoury carried out liquid-phase TERS measurements at a solid–liquid interface using gold nanoplates modified with 4-mercaptobenzonitrile (MBN) in water. By mapping the Raman scattering signal from the molecules, he visualized the heterogeneous molecular coverage with an extremely high spatial resolution of below 3 nm. He also captured in situ the local reaction process in which plasmon-induced hydrolysis of MBN generated 4-mercaptobenzoic acid, showing that water is involved not merely as a medium but also as a reactant [105].

#### 3.5 Neutron and X-ray-based interfacial analysis

**Figure 7:**
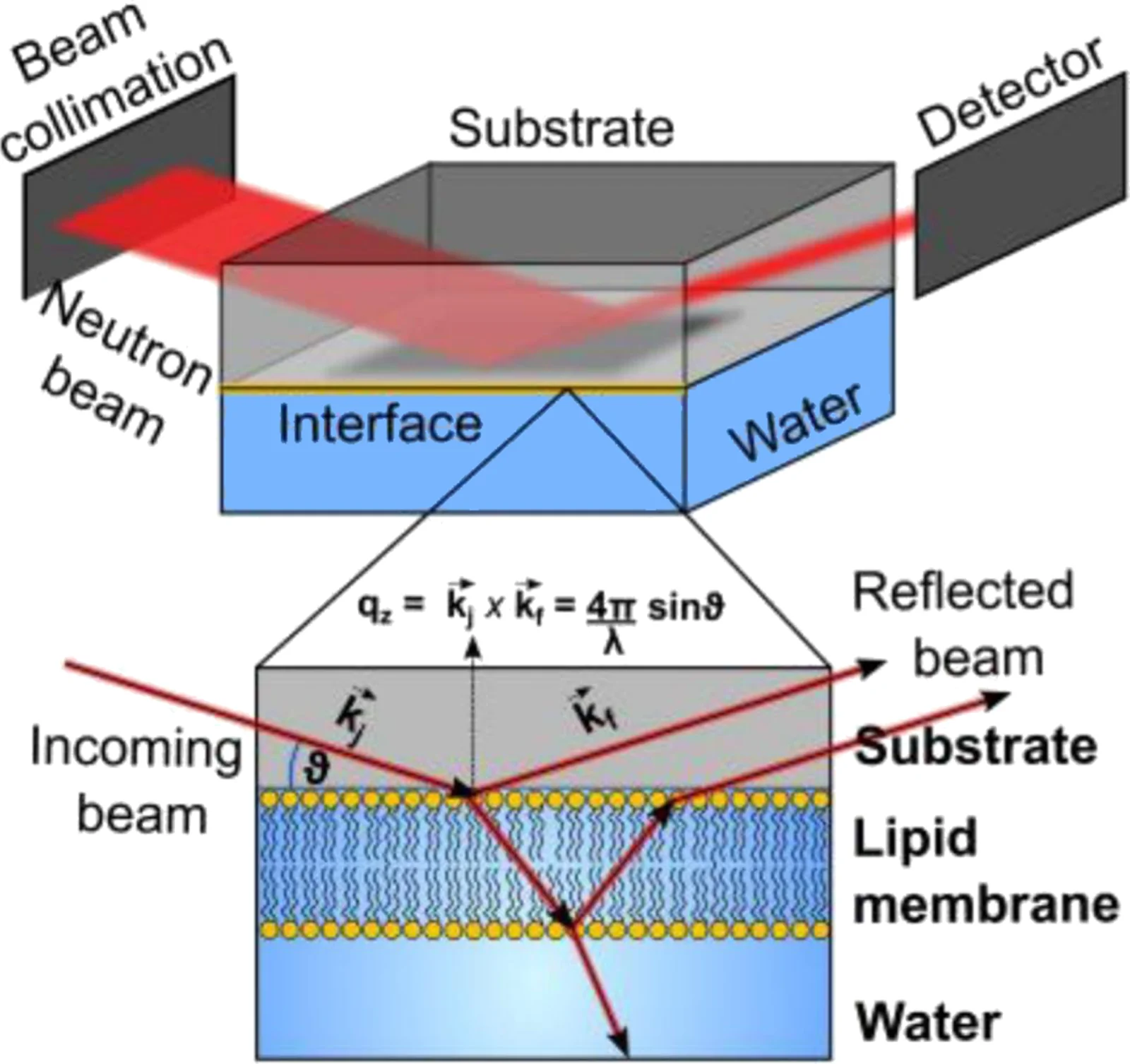
Schematic illustration of the measurement principle for NR. Figure 7 was adapted from [116] (© 2024 L. Caselli et al., published by Elsevier B.V., distributed under the terms of the Creative Commons Attribution 4.0 International License, https://creativecommons.org/licenses/by/4.0/).

**3.5.1 Neutron reflectometry.** Neutron reflectometry (NR) is a technique for evaluating the nanoscale structure of stratified interfaces based on the reflectivity profile of a neutron beam reflected at the interface (Figure 7). A major advantage of this method is its ability to identify specific components at the interface using contrast variation through isotopic substitution [109]. By deriving the scattering length density profile from the measured reflectivity profile, it is possible to evaluate the surface mass density at the interface, the hydration state directly reflecting the spatial distribution of specific components, molecular orientational changes, and even heterogeneous multilayer structures in the thickness direction [110–111]. Pan and co-workers analyzed the self-assembled structure of polypeptoid brushes at the SiO_2_–water interface using in situ NR. By carefully examining the volume fraction profiles obtained through contrast variation with deuterium substitution, they identified a two-tier heterogeneous structure consisting of a dense inner layer containing adhesive units tightly bound to the solid surface, and a highly hydrated, low-density outer layer. They demonstrated that this unique hydration structure contributes to the suppression of protein denaturation and provides excellent antifouling effects [110].

**3.5.2 X-ray reflectometry and X-ray absorption spectroscopy.** Similar to NR, X-ray reflectometry (XRR) is a technique capable of evaluating the nanoscale structure of stratified interfaces from the reflectivity profile of X-rays reflected at the interface. It has the advantage of more easily yielding high-resolution results than NR due to the overwhelming brilliance of synchrotron X-rays [112]. Based on this high resolution, an electron density profile (EDP) representing the spatial distribution of electrons in the material is derived from the measured reflectivity profile. This allows for the evaluation of surface mass density, the hydration state of adsorbed molecular layers, molecular orientation, and multilayer structures in the thickness direction [113–114]. In contrast, X-ray absorption spectroscopy (XAS) is a technique that evaluates the bonding states of molecules present near the substrate interface, such as those in the electric double layer, by analyzing the microscopic electronic components contained in surface-sensitive secondary electrons emitted upon X-ray irradiation. It also has the advantage of selectively extracting the bonding states of molecules by tuning the irradiation energy to specific electron orbitals. By leveraging these characteristics, it is possible to directly evaluate the microscopic bonding states of molecules in response to applied voltages [115]. Thus, while XRR evaluates the electron density distribution and the structure in the thickness direction of the layer formed at the interface, XAS can selectively evaluate the bonding states of molecules present near the interface. Therefore, both are X-ray measurement techniques capable of analyzing solid–liquid interfaces from different perspectives. Richter and co-workers evaluated the adsorption structure and formation process of proteins at the SiO_2_–water interface using in situ XRR. By applying the average mass-to-electron-density ratio of amino acids, they calculated the net adsorbed mass at the interface from the EDP. This approach allowed them to capture the adsorption structure not merely as a static density distribution, but as a dynamic process of increasing adsorbed mass over time. Furthermore, with a spatial resolution superior to that of neutron reflectometry, they demonstrated that proteins adsorb in a highly hydrated, native state and form a dynamically heterogeneous structure in which the density distribution evolves over time to maximize interfacial interactions [114].

**Figure 8:**
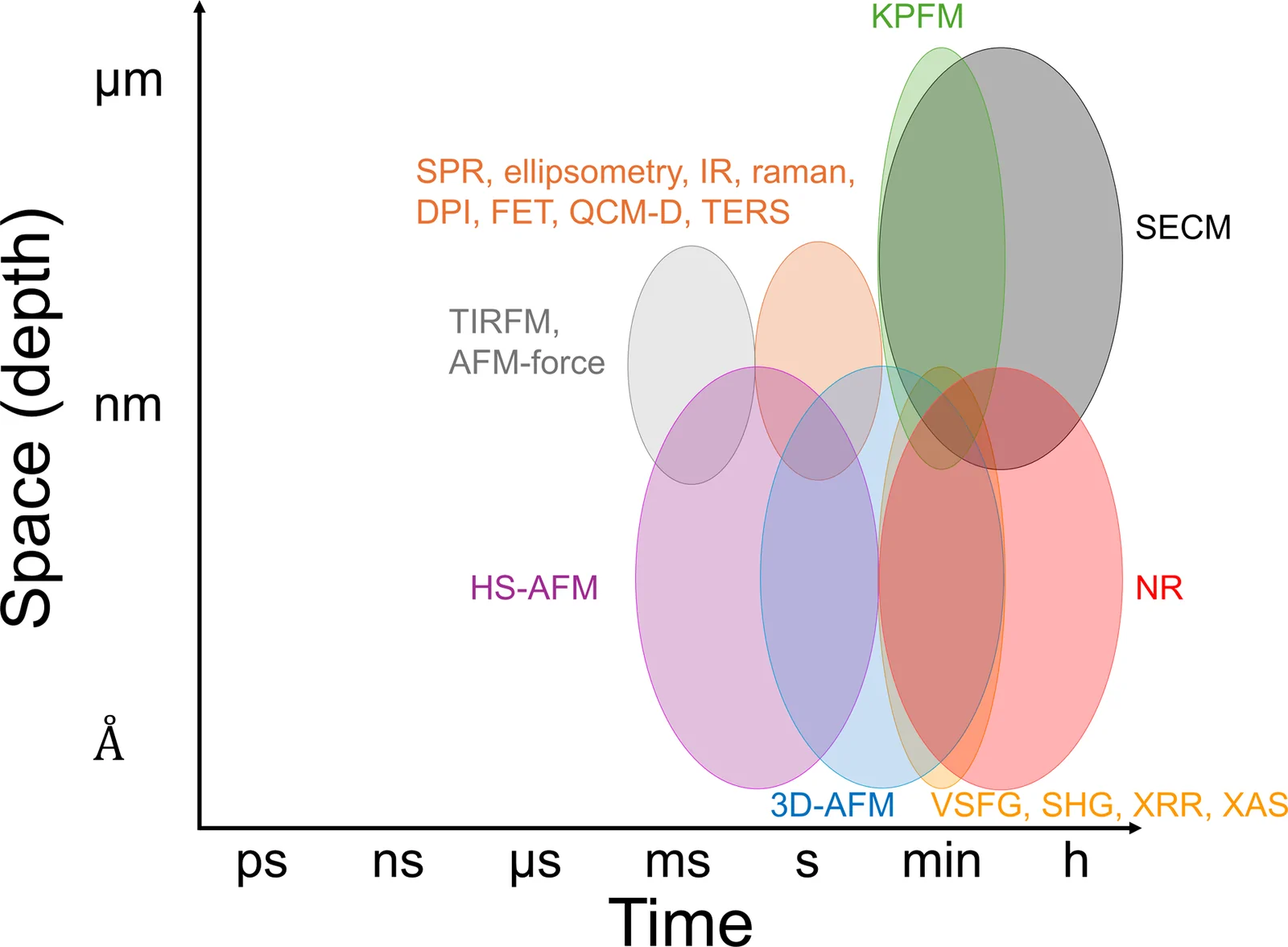
Spatiotemporal mapping that organizes the depth‑direction spatial resolution and standard temporal resolution of stand‑alone methods.

As discussed above, each stand-alone method provides access to only a limited part of the interfacial state because its observation window is restricted by its physical principle. Consequently, no single technique can fully capture the coupled changes in mass, charge, molecular structure, hydration, and local morphology that govern solid–liquid interfacial phenomena. To facilitate a direct comparison, Figure 8 illustrates the spatiotemporal mapping of these stand-alone methods, highlighting their standard temporal and depth-direction spatial resolutions. Additionally, Table 1 summarizes their key characteristics, including the observable physical quantities and their inherent limitations discussed in Sections 3.1–3.4. Based on this comparison, the subsequent sections discuss how multimodal measurement platforms integrate complementary methods to overcome these limitations and provide a more comprehensive view of interfacial dynamics.

**Table 1:** Summary of stand-alone techniques for the characterization of solid–liquid interfaces.

Classification	Technique	Target physical quantity	Target interfacial phenomena	Ref.

optical	SPR	mass	binding kinetics	[9,57]
	Ellipsometry	mass	binding kinetics	[59–60]
	IR	molecular vibration	structural changes, orientation, Intermolecular interactions	[37,61–62]
	TIRFM	number of adsorbed molecules	single-molecule binding kinetics, microfluidic dynamics	[63–65]
	Raman/SERS	molecular vibration	structural changes, orientation, Intermolecular interactions	[67–69]
	VSFG	molecular vibration	structural changes, orientation, hydrogen-bonding network	[14,71–72]
	SHG	charge state, interfacial potential	charging behavior, acid-base equilibrium process	[74–75]
	DPI	mass	structural changes	[77–78]

electrochemical	EIS	charge state, interfacial potential	structural changes in the EDL	[84]
	FET	charge state, interfacial potential	binding kinetics, charge rearrangement	[18,86–87]
	SECM	electrochemical properties, local charge state	local electron transfer processes, electrostatic interactions	[41,89]

mechanical	QCM-D	hydrated mass, viscoelasticity	binding kinetics, hydration state, structural changes	[12,51,92]
	AFM-force	local mechanical properties, interaction forces	hydration state, structural changes	[93–94]

SPM	AFM	local structure	structural changes	[95–96]
	FM-AFM/3D-AFM	interaction forces, local structure	hydration structure	[98–100]
	KPFM	local interfacial potential	charging behavior, screening effect of the EDL	[101]
	TERS	local molecular vibration	structural changes, orientation, chemical reaction processes	[103–105]

neutrons and X-ray	NR	density profiles	hydration state, orientation, multilayered structure	[110–111]
	XRR	density profiles	hydration state, orientation, multilayered structure	[113–114]
	XAS	local bonding states	structural changes, orientation, hydrogen-bonding network	[115]

### 4 Advances in multimodal characterization techniques

#### 4.1 Integration of optical methods with electrochemistry (EC)

**4.1.1 SPR–EC systems.** SPR–EC systems combine interfacial electrochemical readouts with local refractive-index responses from the same interface, thereby enabling direct comparison between charge-transfer processes and concomitant changes in adsorbed mass or effective film thickness [117–124]. The Kretschmann geometry, in which the metal film on the base of a prism serves as the working electrode, has been the standard optical configuration [118–120,122–124]. More recently, voltage-modulated normal-incidence SPR based on gold nanohole arrays that do not require a prism has also been developed to facilitate device miniaturization and integration with microfluidic channels [117,121].

**Figure 9:**
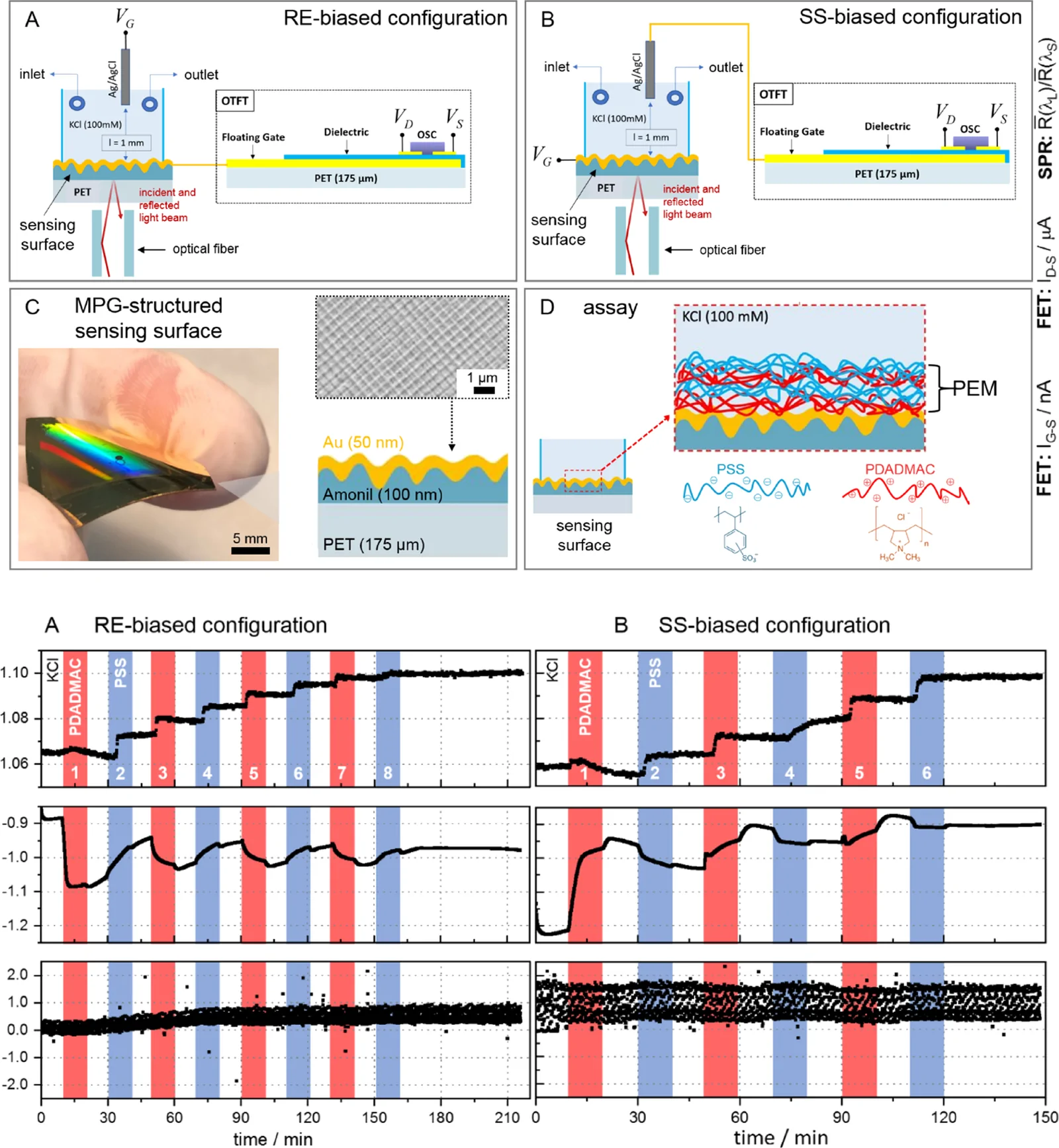
Representative multimodal measurement platforms integrating electrochemical control with optical measurements, together with representative outputs: SPR–EC platform used for polyelectrolyte sensing. Figure 9 was reproduced from [117] (© 2025 D. Hatami et al., published by Springer Nature, distributed under the terms of the Creative Commons Attribution-NonCommercial-NoDerivatives 4.0 International License, https://creativecommons.org/licenses/by-nc-nd/4.0/). This content is not subject to CC BY 4.0.

Hatami and co-workers developed a hybrid sensor that integrates an extended-gate organic thin-film transistor (ExG-OTFT) with SPR and improved system stability by spatially separating the sensing surface from the transistor body. From simultaneous measurements during the formation of polyelectrolyte multilayers, they showed that SPR captures local refractive-index changes associated with mass adsorption, whereas the ExG-OTFT captures the collective distribution of charge carriers over the whole interface, thereby demonstrating the complementary nature of the information obtained [117] (Figure 9). Zhao and co-workers also constructed a device for the simultaneous measurement of cellular exocytosis by combining an UME with SPR, and used K^+^ stimulation to trigger exocytosis in PC12 cells cultured on a gold thin film. The carbon nanopipette electrode detected small current spikes from dopamine released from single cells, while SPR simultaneously recorded macroscopic refractive-index changes caused by fusion of vesicles with the cell membrane, revealing a clear positive correlation between the SPR signal and the frequency of exocytotic events [118].

Technical limitations nevertheless remain. When a gold thin film serves as the working electrode, potential-dependent changes in surface electron density can introduce background shifts in the SPR response, which may obscure the redox-related signal of the target molecules [122]. In addition, the two channels do not necessarily probe the same spatial domain: In ExG-OTFT–SPR systems, the electrical signal reflects charge redistribution over the entire electrode, whereas SPR monitors local refractive-index changes only within the illuminated region. Consequently, delayed molecular transport within the flow cell can generate an apparent time lag between the two signals [117]. In more complex systems such as cellular exocytosis, the SPR response may also include contributions from cell-membrane deformation and bulk refractive-index changes, complicating rigorous quantification of released molecules [118,120].

Taken together, these examples show that the main advantage of SPR–EC systems is the ability to relate charge-transfer events to changes in adsorbed mass, effective film thickness, or interfacial refractive index, rather than measuring each response independently. However, this correlation can be complicated by potential-induced optical background shifts, differences in the effective sensing areas of the two channels, mass-transport delays within flow cells, and non-specific refractive-index contributions in complex biological samples. Therefore, future SPR–EC platforms should define the spatial, temporal, and physicochemical relationship between optical and electrochemical responses more rigorously, while improving the sensitivity of each measurement channel.

**4.1.2 SERS–EC systems.** SERS–EC systems, which integrate electrochemical measurements with surface-enhanced Raman scattering, are analytical platforms that enable real-time analysis of complex reaction dynamics at the molecular level by simultaneously measuring electrochemical responses at an interface and molecular vibrational signals reflecting the chemical structure and orientation of adsorbed molecules at the same interface [21–22,125–127]. The standard optical configuration employs roughened metal surfaces as the working electrode [21–22,125], but more recently shell-isolated nanoparticle-enhanced Raman spectroscopy, in which plasmonic nanoparticles are coated with an ultrathin inert silica shell, has also attracted attention [126–127]. Collectively, these approaches enable dynamic control of adsorbate orientation through potential modulation and highly sensitive operando measurements of biomacromolecules such as DNA and other complex organic molecules.

As a specific example, Wicaksono and co-workers used a CV-SERS device based on electrodeposited gold nanostructures on a screen-printed carbon electrode to simultaneously track the electrochemical oxidation of malachite green (MG) and changes in its adsorbate orientation. Analysis of the intensity ratio of specific peaks showed that under positive potentials MG forms dimers and adsorbs strongly in a tilted upright configuration through its aromatic rings [22]. Tran and co-workers further developed an SERS–EC method in which the surface potential was dynamically modulated during measurements of DNA oligonucleotides, thereby expanding the information that could be extracted from potential-dependent reorientation. By pulling different bases into the hotspot at different potentials and combining the resulting spectra from multiple conformations into a “superprofile”, together with machine learning, they identified DNA base composition, strand length, and primary sequence with high accuracy [21].

Several technical limitations are also characteristic of SERS–EC systems. Potential modulation can alter the electronic environment at the metal surface and induce Raman peak shifts through the electrochemical Stark effect, so spectral changes must be interpreted with caution [125]. Under harsh electrochemical or catalytic conditions, electrode reconstruction or nanoparticle reshaping can further change hotspot distributions and destabilize signal intensity [128]. In addition, for large molecules such as DNA, the surface selection rule preferentially enhances signals from segments located close to the substrate, so the measured spectra may represent only part of the molecular structure [125].

These studies demonstrate that SERS–EC is particularly useful for linking electrochemical potential control with molecular-level vibrational responses at plasmonic electrode interfaces, which cannot be obtained from current–potential measurements alone. At the same time, the main issue in SERS–EC systems is not only signal enhancement, but also the reliable assignment of potential-dependent spectral changes. Because SERS spectra can be influenced by adsorbate orientation, local electric fields, hotspot geometry, and surface-selection effects, apparent spectral changes do not always correspond directly to chemical transformation at the interface. Future SERS–EC studies should therefore combine stable plasmonic electrode designs with analytical strategies that distinguish true molecular changes from field-, orientation-, and geometry-dependent spectral variations.

**4.1.3 IR–EC systems.** IR–EC systems, which integrate electrochemical measurements with IR spectroscopy, are analytical platforms that enable molecular-level analysis of complex reaction dynamics by correlating electrochemical responses at an interface with molecular vibrational signals that reflect the chemical structure, hydrogen-bond network, and orientation of adsorbed molecules [128–130]. Methods tailored to the target system have been developed, including SEIRAS, which uses plasmonic field enhancement [129], polarization-modulation infrared reflection absorption spectroscopy (PM-IRRAS), which suppresses bulk-water absorption by polarization modulation [130], and polarized ATR methods that use orthogonal polarizations (p and s) to analyze molecular orientation with high precision [128]. As a result, applications have expanded broadly across organic and biological systems, from the basic behavior of monolayers to tracking antimicrobial mechanisms using model cell membranes and evaluating liposome-based drug-delivery systems.

This capability to evaluate and control interfacial molecular orientation using potential as a trigger has also been used to elucidate the behavior of complex biomolecular systems such as amino-acid residues and cell membranes. Pudžaitis and co-workers focused on the histidine side chain, which plays an important role in protein structure and function, and prepared an imidazole-terminated SAM on a gold electrode for SEIRAS analysis. By following the low-wavenumber shift of the imidazole =C–H stretching modes (3115 and 3150 cm^−1^) when the potential was changed toward more negative potentials (from 0.3 to −0.5 V), they demonstrated at the molecular level that negative electrode polarization reversibly weakens hydrogen bonding of imidazole at the organic layer/water interface [129]. Extending this concept to a more complex membrane model, Su and co-workers constructed on a gold electrode a model lipid bilayer mimicking the outer membrane of Gram-negative bacteria (Lipid A and 1,2-dimyristoyl-*sn*-glycero-3-phosphoethanolamine) and tracked its interaction with the antibiotic polymyxin by operando PM-IRRAS. Analysis of specific peaks quantitatively revealed the process by which, under an applied membrane potential, polymyxin competes with Mg^2+^ to bind to polar headgroups, increases the tilt angle of lipid acyl chains, changes the orientation of C=O bonds, and thereby disrupts the membrane structure [130].

IR–EC systems also face several common interpretational challenges. In metal-enhanced configurations, potential-induced peak shifts may reflect either genuine changes in the local chemical environment, such as hydrogen-bond reorganization, or electric-field-induced Stark effects, and these contributions are not always easy to distinguish. Harsh potential cycling may also damage or delaminate the metal thin film used for signal enhancement [129]. In aqueous measurements using PM-IRRAS and related methods, variations in optical geometry, such as the thickness of the water gap, can strongly affect signal intensity, making rigorous normalization essential for quantitative comparison [130]. Furthermore, in complex biomimetic membranes or drug–membrane systems, absorption bands from different components often overlap, which complicates spectral assignment and quantitative interpretation [128,130].

The reviewed IR–EC studies show that coupling infrared spectroscopy with electrochemical control makes it possible to follow potential-dependent changes in interfacial molecular structure, hydrogen-bonding states, and molecular orientation that cannot be resolved from electrochemical responses alone. In particular, these systems provide vibrational evidence for how adsorbed species, hydrogen-bonding networks, and membrane components reorganize under applied potentials, thereby extending electrochemical analysis from charge-transfer behavior to molecular-level structural interpretation. However, the main interpretational difficulty in IR–EC systems arises from the fact that vibrational bands are strongly affected by hydrated and compositionally complex interfacial environments. Changes in hydrogen-bonding networks, electric-field effects, optical geometry, and overlapping absorption bands can produce similar spectral variations, making it difficult to assign a given IR response to a single molecular process [113–115]. Future IR–EC studies should therefore combine carefully controlled optical and electrochemical configurations with isotope labeling, spectral deconvolution, and computational support to distinguish genuine interfacial molecular rearrangements from hydration-, field-, geometry-, and composition-dependent spectral variations.

**4.1.4 VSFG–EC systems.** VSFG–EC systems enable real-time, molecular-level analysis of complex reaction dynamics by simultaneously measuring electrochemical responses and nonlinear optical signals at the same interface, where the latter reflect the local orientation, chemical structure, and interfacial electric field of adsorbed molecules [131–134]. Applications of this system have expanded from high-precision mapping of local interfacial electric-field profiles using the vibrational Stark effect [132–134] to real-time tracking of trace reaction intermediates and their orientation in energy-conversion reactions [131].

This ability to evaluate molecular orientation and local electric fields at interfaces has also been applied to the clarification of the adsorption structures and interfacial environments of complex organic molecules. Liu and co-workers used broadband SFG with a polycrystalline copper electrode to simultaneously track the C–H stretching vibrations and dynamic structural changes of ethoxy groups, which are organic reaction intermediates in CO_2_ reduction. Analysis of specific peaks demonstrated that the ethoxy group adopts two distinct adsorption structures, namely, a standing orientation, in which the ethyl group points away from the electrode, and a lying orientation, in which the methyl group lies very close to the surface [131]. Ge and co-workers further constructed SAMs of aromatic diisocyanide molecules with different chain lengths on gold electrodes and combined SFG-based measurements of the interfacial electric field with MD simulation. They quantified that short molecules form tightly packed layers, whereas longer and more flexible molecules leave intermolecular gaps that allow water and electrolyte ions to penetrate into the organic film (percolation), thereby changing the effective thickness of the EDL [133].

VSFG–EC systems have their own technical limitations as well. On metal electrodes, nonresonant background signals can interfere with resonant vibrational features, and phase changes associated with surface reconstruction may even invert the spectral line shape, making purely chemical interpretation difficult [131]. Under Faradaic conditions, the local interfacial electric field does not always scale linearly with the applied voltage; instead, “leaky capacitor” behavior may produce a mismatch between the applied potential and the actual molecular polarization [132]. In realistic catalytic environments, additional complications arise from solvent and ion penetration into the interfacial film and from hydrogen-bonding interactions with probe molecules, both of which can perturb vibrational frequencies and electric-field estimates [132–133].

By resolving nonlinear vibrational responses under electrochemical control, VSFG–EC studies have shown that potential-dependent changes in interfacial molecular orientation, ordering, and local electric fields can be analyzed beyond electrochemical responses alone. However, in contrast to linear vibrational spectroscopies, VSFG–EC measurements require particular care in separating genuine chemical dynamics from electrostatic and optical contributions in nonlinear interfacial spectra. Because VSFG responses are sensitive not only to molecular structure and orientation, but also to local electric fields, nonresonant background interference, and phase changes associated with metal-surface reconstruction, potential-dependent spectral variations do not necessarily map directly onto chemical transformations [117–120]. Future VSFG–EC studies should therefore combine controlled electrochemical measurements with phase-sensitive line-shape analysis and computational modeling to distinguish genuine interfacial molecular dynamics from electric-field- and optics-dependent spectral variations.

**4.1.5 Time-resolved spectroscopy–EC systems.** Systems that integrate electrochemistry with time-resolved spectroscopy provide a platform for tracking ultrafast charge transfer and short-lived intermediates at interfaces. Methodologically, they can be broadly divided into ultrafast spectroscopy triggered by photoexcitation and operando measurements triggered by potential perturbation [135–139]. The former includes pump–probe methods and two-dimensional electronic spectroscopy (2DES) and two-dimensional infrared spectroscopy using ultrashort pulsed lasers [135,137,139], which follow ultrafast responses immediately after excitation under potentiostatic control. The latter includes methods such as rapid-scan ATR-SEIRAS [136], which can follow transient responses after a potential step on a timescale of tens of milliseconds. These approaches have made it possible to elucidate highly diverse interfacial dynamics, including heterogeneous structural dynamics of molecules at electrode interfaces, molecular reorientation immediately after a potential step, and real-time tracking of electrochemical grafting on carbon electrodes [127–128,130].

**Figure 10:**
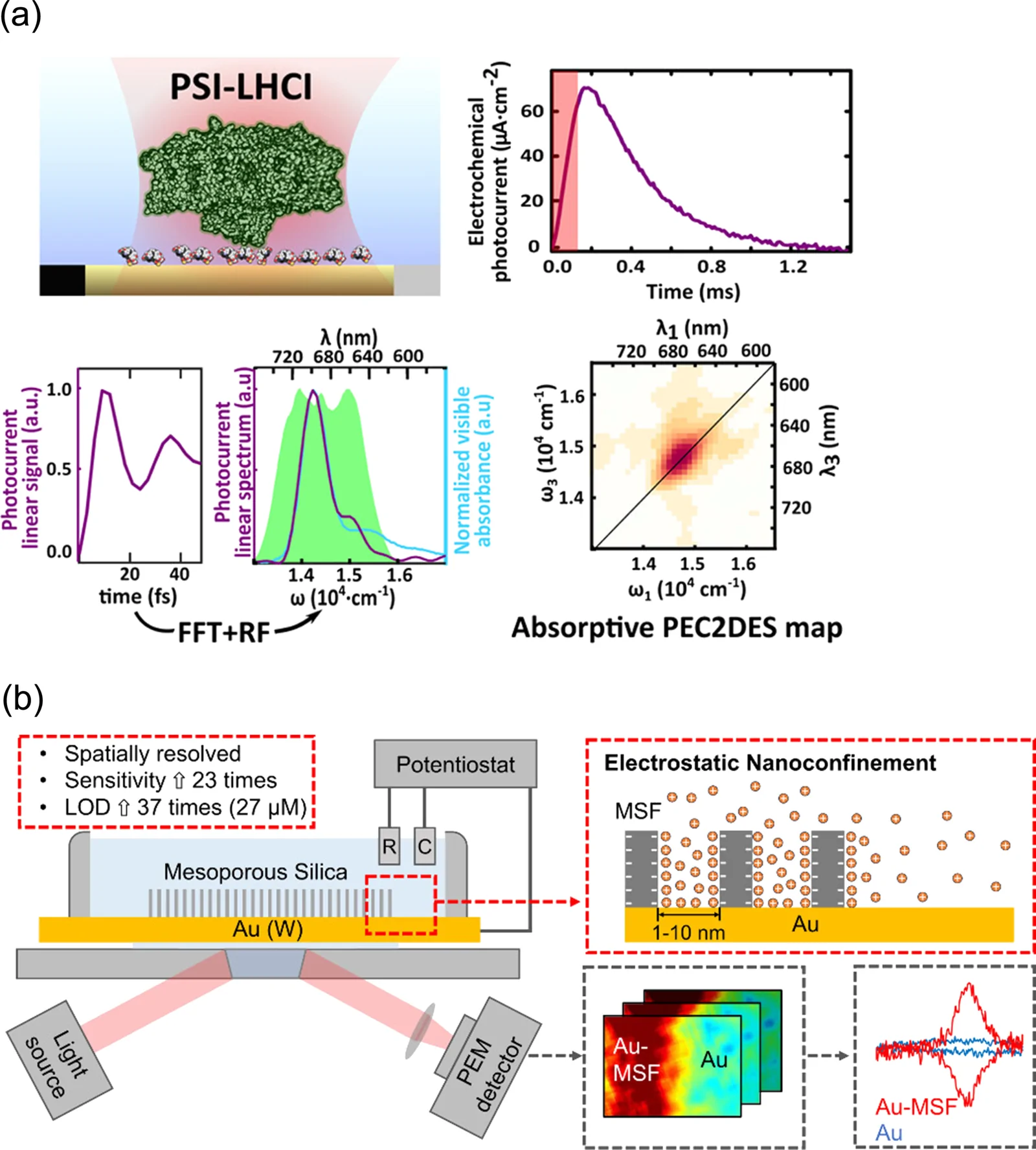
Representative multimodal measurement platforms integrating electrochemical control with optical measurements, together with representative outputs: (a) EC–2DES platform used for functional analysis of photosystem I. Figure 10a was reproduced from [139] (© 2024 M. López-Ortiz et al., published by American Chemical Society, distributed under the terms of the Creative Commons Attribution-NonCommercial-NoDerivatives 4.0 International License, https://creativecommons.org/licenses/by-nc-nd/4.0/). This content is not subject to CC BY 4.0. (b) MSF-modified PEM platform used for dopamine visualization. Figure 10b was reproduced from [140] (© 2025 S. Groysman et al., published by American Chemical Society, distributed under the terms of the Creative Commons Attribution-NonCommercial-NoDerivatives 4.0 International License, https://creativecommons.org/licenses/by-nc-nd/4.0/). This content is not subject to CC BY 4.0.

These advanced time-resolved multimodal measurements have proven powerful for elucidating biohybrid photoelectrodes based on photosynthetic proteins and the charge-transfer dynamics of organic monolayers. Nawrocki, López-Ortiz, and their co-workers carried out simultaneous time-resolved spectroscopy and photocurrent measurements in systems where photosynthetic protein complexes (reaction center–light-harvesting 1 (RC-LH1) and photosystem I–light-harvesting complex I (PSI-LHCI)) were immobilized on electrodes. This enabled quantitative identification of bottlenecks in electron transfer that inhibit photoinduced charge separation and of energy-loss pathways caused by short-circuiting to the electrode. By further extending this approach to photoelectrochemical two-dimensional electronic spectroscopy, they selectively extracted exciton dynamics that truly contribute to charge separation from the complicated spectra of densely packed pigments [137,139] (Figure 10a). As a simpler interfacial system involving organic molecules, Unni and co-workers constructed a SAM of the stable 2,2,6,6-tetramethylpiperidine-1-oxyl (TEMPO) radical on a gold island film and used time-resolved ATR-SEIRAS. By following the spectra with a high time resolution of 40 ms, they dynamically captured the reversible conformational change (chair inversion) of the piperidinyl ring accompanying the redox process of TEMPO and demonstrated at the molecular level that this conformational change contributes to asymmetric electron-transfer kinetics [136].

Time-resolved spectroscopy–EC systems also face several method-specific challenges. In ultrafast measurements, intense pulsed-laser irradiation can damage both samples and electrode surfaces, making careful control of excitation intensity essential [135]. In two-dimensional spectroscopy of multichromophoric systems, exciton–exciton annihilation and contamination by linear signals (incoherent mixing) can complicate the extraction of genuine energy-transfer and coherence dynamics [139]. In infrared-pulse-based measurements, additional background contributions may arise from free-carrier excitation in semiconductor substrates [135]. Moreover, because electrochemical current reflects a macroscopic response whereas optical readouts often probe a local region, spatiotemporal mismatches can arise between the two channels [136]. In some photoelectrochemical systems, comparison of spectroscopic and electrochemical responses has even enabled short-circuit losses to be quantified [137].

By combining time-resolved spectroscopy with electrochemical measurements, these systems have made it possible to relate transient photoinduced molecular events to subsequent charge separation, transfer, and extraction processes that cannot be resolved from steady-state electrochemical responses alone. For time-resolved spectroscopy–EC systems, the main difficulty lies in determining how transient molecular events captured optically are translated into measurable electrochemical outputs. Ultrafast or local spectroscopic signals do not necessarily represent charge carriers that are ultimately extracted as current, because optical artifacts, kinetic heterogeneity, and device-dependent losses can decouple the spectroscopic response from the electrochemical output [121–125]. Future platforms should therefore be designed to connect transient optical signatures more directly with electrochemical charge-extraction pathways through precise synchronization, optimized excitation conditions, and cell or substrate architectures that minimize such decoupling.

**4.1.6 Nanoconfinement-assisted optical–electrochemical systems.** As an example of a signal-amplified optical–electrochemical platform, plasmonic electrochemical microscopy (PEM) modified with a mesoporous silica film (MSF) uses electrostatic nanoconfinement within surface pores to locally enrich oxidized species and thereby enhance the optical signal [140]. This makes it possible to compensate for the limited sensitivity of conventional PEM and to visualize low-concentration species in microscopic regions in real time under operando conditions.

Using an Au–MSF electrode, Groysman and co-workers demonstrated a highly sensitive plasmonic imaging–electrochemistry platform in which nanoconfinement amplified the local concentration of electroactive species. With 1,1′-ferrocenedimethanol (FC) as a model analyte, confinement of oxidized FC^+^ within the pores improved the detection limit up to 37-fold and the sensitivity 23-fold relative to standard PEM. The system also enabled visualization of dopamine oxidation following local delivery from a micropipette, illustrating its potential for real-time imaging of neurotransmitter release [140] (Figure 10b). This method nevertheless has several practical limitations. Nonuniform deposition of the MSF can generate spatial fluctuations in the PEM signal, and conversion of the optical response into absolute current or concentration remains difficult. In addition, differences between the concentration at the source and that at the confined surface complicate rigorous quantitative interpretation [140].

Although nanoconfinement provides a powerful strategy for amplifying optical–EC signals, this example also emphasizes that improved optical sensitivity must be accompanied by careful quantitative correlation with electrochemical processes. This requirement is common to the optical–EC systems reviewed in this section, in which electrochemical control is linked with optical information on interfacial mass transport, molecular orientation, bonding states, local electric fields, and transient reaction intermediates. Future optical–EC platforms should therefore be designed to correlate optical and electrochemical responses through controlled device architectures, appropriate calibration, synchronized measurements, and physically informed analysis.

#### 4.2 Integration of optical methods with QCM-D

**4.2.1 SPR–QCM-D systems.** SPR–QCM-D systems integrate QCM-D with SPR-based techniques and serve as powerful analytical platforms that combine two different physical principles, acoustic waves and electromagnetic waves, to measure complex dynamics at the same interface in real time [12,20,141–147]. Their configurations range from approaches that evaluate the same model interface in parallel with independent instruments [12,20,141,144–147] to approaches that integrate both probes on a single sensor substrate, such as a quartz chip patterned with metallic nanostructures, and perform truly simultaneous in situ measurements in the same flow cell [142–143]. The greatest advantage of this method is that the hydration ratio of the adsorbed layer can be estimated from the difference between the “wet mass”, including solvent, measured by QCM-D and the optically estimated “dry mass” or thickness obtained by SPR/LSPR, thereby providing important information on biological interfaces that cannot be obtained by either technique alone [12,20,147].

Asai and co-workers constructed a LSPR–QCM-D sensor based on an anodic aluminum oxide nanohole array and evaluated the process from adsorption of giant liposomes (diameter about 220 nm) to local membrane deformation and disruption induced by exposure to the surfactant Triton X-100. Correlation analysis of resonance-frequency change, resonance resistance change, and LSPR resonance-wavelength shift revealed the interfacial response after liposome adsorption into a series of stepwise processes, that is, membrane deformation, swelling due to micelle interaction, membrane rupture accompanied by leakage of the internal solution, and finally lipid-bilayer formation [143]. Komorek and co-workers used analysis of the difference between the wet mass obtained by QCM-D and the dry mass obtained by SPR to show that the orientation of lysozyme adsorbed on a gold surface shifts from a side-on configuration to an end-on configuration as surface coverage increases. They also showed that the hydration ratio of this adsorbed layer changes with pH from about 70% at pH 4.0 to about 30% at pH 11.0 [20]. Yoon and co-workers further analyzed lysozyme adsorption in water–ethanol mixed solvents and showed that ethanol concentration non-monotonically affects densification of the adsorbed layer and the amount of hydration through changes in protein structural flexibility. In approximately 30% ethanol, pronounced densification of the adsorbed layer and dehydration were observed [147].

At the same time, significant technical limitations remain. Arrizabalaga and co-workers quantitatively demonstrated that in highly concentrated salt solutions, sensor signals are strongly governed by changes in the viscosity, density, and refractive index of the bulk liquid, making it difficult to separate these bulk effects from the true adsorption signal [146]. They also noted that as a basic limitation of the method, QCM-D essentially lacks an internal reference channel, so the reliability of the data becomes severely limited in systems where solution properties change substantially [146]. In addition, as noted in the analyses of several studies, choosing the wrong model, for example, between the Voigt and Sauerbrey models, and overlooking local heterogeneity because of macroscopic spatial averaging can also become general sources of error [12,144,146].

In SPR–QCM-D systems, comparison of the optical “dry” mass with the acoustic “wet” mass makes it possible to interpret their discrepancy not merely as an analytical limitation, but as a key source of information on hydration, viscoelasticity, molecular orientation, and interfacial heterogeneity [12,20,129,133]. However, extracting this information requires careful separation of true interfacial adsorption and restructuring from bulk-liquid effects, model-dependent QCM-D analysis, optical assumptions, and spatial averaging over heterogeneous interfaces [12,130,132]. Future platforms should therefore emphasize controlled solution conditions and coupled optical–acoustic modeling to quantitatively resolve hydration and viscoelastic contributions to interfacial processes.

**4.2.2 SERS–QCM-D systems.** SERS–QCM-D systems integrating QCM with SERS provide an analytical platform that acquires, in a complementary manner from the same interface, macroscopic changes in mass and viscoelasticity together with microscopic molecular vibrational spectra and chemical information [148–151]. The greatest strength of this approach lies in combining the quantitative adsorption-mass information from QCM with the molecular “chemical fingerprint” provided by SERS, thereby enabling simultaneous access to information that is in principle unattainable by either technique alone. Instrumentally, these systems can be divided into two principal configurations, namely, fully in situ simultaneous measurements, in which plasmonically active nanostructures are formed directly on the QCM electrode and measured under laser irradiation [148–150], and tandem measurements, in which the electrode is transferred to a Raman microscope after QCM measurements, often using SERS nanotags [151].

**Figure 11:**
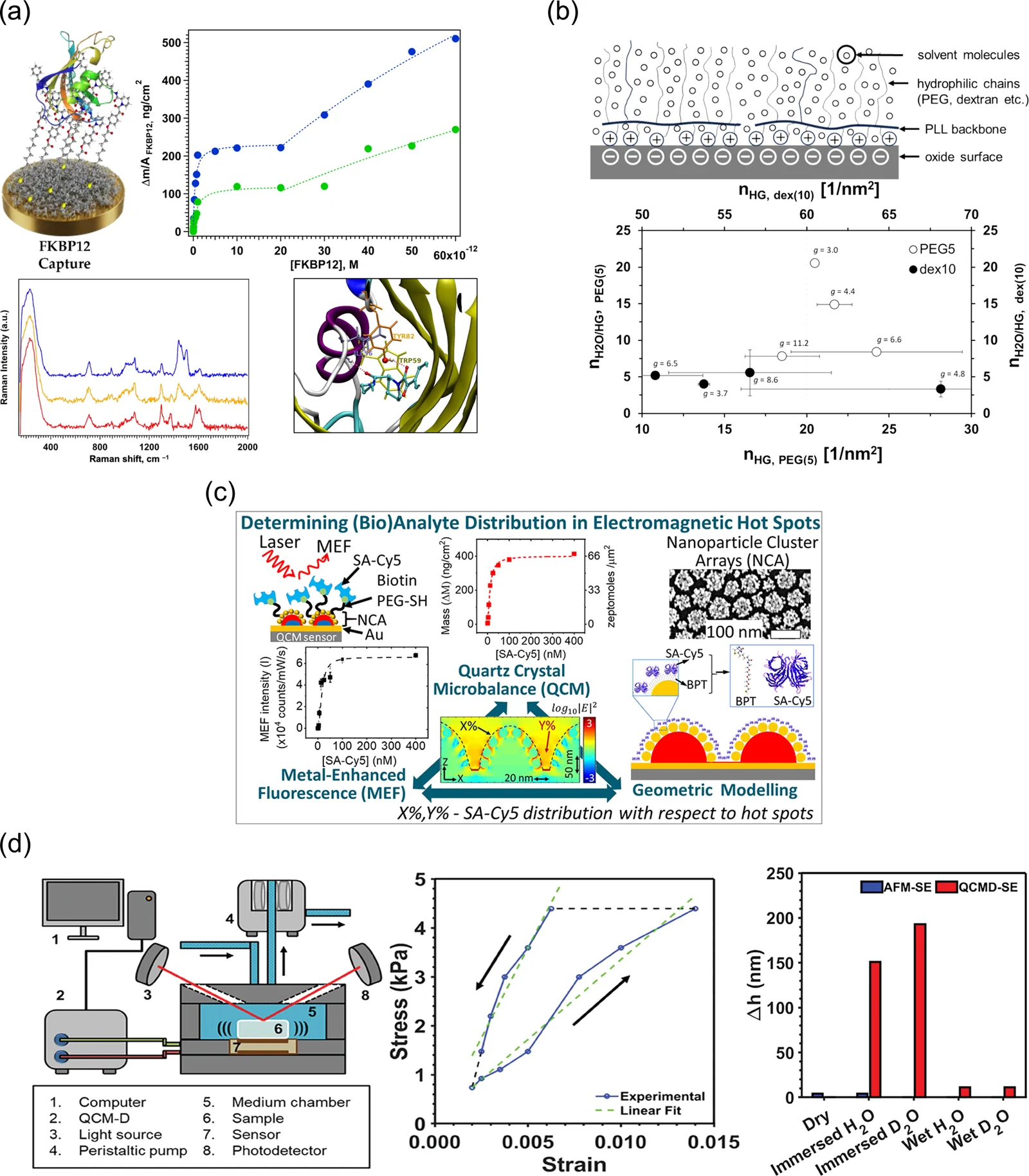
Representative multimodal solid–liquid interface measurement platforms that integrate optical probes with QCM measurements, together with representative outputs. (a) QCM–SERS platform used for FKBP12 detection. Figure 11a was adapted from [148] (© 2025 C. Bartolini et al., published by MDPI, distributed under the terms of the Creative Commons Attribution 4.0 International License, https://creativecommons.org/licenses/by/4.0/). (b) OWLS–QCM platform used for quantifying hydration of polymer brushes. Figure 11b was adapted from [152] (© 2024 C. Perrino et al., published by American Chemical Society, distributed under the terms of the Creative Commons Attribution 4.0 International License, https://creativecommons.org/licenses/by/4.0/). (c) MEF–QCM platform used for analysis of SA–Cy5 molecular distribution. Figure 11c was reproduced from [153] (© 2021 R. Rastogi et al., published by American Chemical Society, distributed under the terms of the Creative Commons Attribution-NonCommercial-NoDerivatives 4.0 International License, https://creativecommons.org/licenses/by-nc-nd/4.0/). This content is not subject to CC BY 4.0. (d) SE–QCM platform used for evaluating the physical properties of ultrathin hydrogels. Figure 11d was adapted from [154] (© 2024 J. Sans et al., published by Wiley-VCH GmbH, distributed under the terms of the Creative Commons Attribution 4.0 International License, https://creativecommons.org/licenses/by/4.0/).

Lequeux and co-workers used a dual sensor patterned with a gold nanocylinder array to simultaneously evaluate the binding of DNA aptamers and the small-molecule antibiotic streptomycin by QCM-D and SERS. QCM-D quantified the dissociation constant (*K*_D_ = 23 nM) and binding stoichiometry, while SERS captured in situ local structural changes, with specific bases within the aptamer (adenine and guanine) showing Raman-band shifts and decreased intensity upon target recognition [150]. Bartolini and co-workers further used a sensor electrode coated with silver nanodendrites by electrodeposition to quantify FKBP12 binding by QCM with a limit of detection of 0.2 pM, while simultaneously detecting receptor conformational changes by SERS, such as reduced vibrational freedom of amide and aromatic-ring modes [148] (Figure 11a). Armero and co-workers captured prostate-specific antigen with an aptamer, quantified the mass by QCM-D, and then introduced three lectin-modified SERS nanotags to multiplex cancer-specific glycan patterns such as fucosylation and sialylation on a single platform [151]. Hou and co-workers used metal-organic frameworks-composite nanofibers to separate and simultaneously quantify chemically similar mixed gases (toluene and benzaldehyde), which cannot be discriminated by QCM alone, over the range of 0–100 ppm by integrating molecule-specific SERS peaks (777 and 820 cm^−1^) with a mathematical algorithm [149].

At the same time, as pointed out in the background sections of these studies, SERS signals alone have serious limitations in quantitation [148–149]. The studies themselves note that nonuniform electromagnetic enhancement at hotspots and variation in molecular orientation constrain quantitative interpretation of SERS signals. Each work addressed this limitation in a different way. Hou and co-workers corrected signal fluctuations using a differential algorithm based on normalized intensities and then combined the results with total mass data from QCM [149], whereas Bartolini and co-workers ensured quantitation using a ratiometric internal standard approach (*I*_1571_/*I*_1438_) [148]. In complex environments, nonspecific adsorption (fouling) can also interfere directly with SERS signals, and, as shown by Armero and co-workers, robust aptamer immobilization strategies such as biotin–streptavidin coupling are indispensable [151].

These limitations indicate that the main value of SERS–QCM-D systems lies in assigning QCM-D-derived adsorption and viscoelastic changes to specific molecular fingerprints, rather than treating them as nonspecific interfacial responses. However, this requires not only quantitative correction of SERS signals, but also careful chemical interpretation of the mass and viscoelastic changes measured by QCM-D. Because QCM-D responses alone cannot identify which molecular species, binding events, or structural rearrangements are responsible for adsorption and mechanical changes, future SERS–QCM-D platforms should emphasize mass-normalized and time-correlated SERS analysis to improve the chemical assignment of QCM-D responses.

**4.2.3 Other optical–QCM-D systems.** In addition to plasmonic methods typified by SPR and SERS, a variety of optical measurements integrated with QCM have been proposed, including ellipsometry, waveguide-mode spectroscopy, and fluorescence enhancement. These methods combine optical and mass responses to obtain interfacial information. In this section, representative examples such as spectroscopic ellipsometry (SE)–QCM, optical waveguide lightmode spectroscopy (OWLS)–QCM, and metal-enhanced fluorescence (MEF)–QCM are briefly reviewed [152–154].

Perrino and co-workers operated QCM-D and OWLS as parallel but non-coincident measurements on equivalent substrates to compare the adsorption behavior of poly(ʟ-lysine)-*graft*-poly(ethylene glycol) (PLL-*g*-PEG) and poly(ʟ-lysine)-*graft*-dextran (PLL-*g*-dex) on oxide surfaces. By quantifying the hydration amount per unit area (Ψ) from the difference between the dry mass obtained by OWLS and the wet mass obtained by QCM-D, they found that Ψ increased with increasing grafting density for both systems; the increase was much larger for PEG, reflecting the greater structural rigidity of dextran. The authors further showed that PEG contains two to three strongly bound water molecules per ethylene glycol unit, forming cage-like structures that contribute to PEG’s excellent antifouling and lubricating properties [152] (Figure 11b).

Rastogi and co-workers used a diblock copolymer template to self-assemble and pattern a gold nanoparticle cluster array on a QCM electrode and then carried out fully in situ simultaneous measurements of QCM and MEF through a microfluidic cell with a sapphire window. By correlating the surface mass density of fluorescently labeled streptavidin (SA-Cy5) obtained by QCM with the enhanced fluorescence intensity from MEF and combining the result with geometric modeling, they quantitatively demonstrated that about 83% of the SA-Cy5 were distributed in the matrix regions between the clusters, where they benefited from electromagnetic enhancement. They also showed that MEF could detect an extremely small amount of SA-Cy5, about 2 zmol, even though the measurement spot was less than one hundred-millionth of the QCM sensing area [153] (Figure 11c).

Sans and co-workers studied ultrathin hydrogel films prepared by plasma polymerization and performed fully simultaneous measurements of QCM-D and SE in a dedicated combined cell. From the difference in thickness measured by the two methods, they successfully separated water hydrated within the film from water trapped at the interface. By deforming the gel with the dynamic pressure of a peristaltic pump and combining the viscoelastic parameters obtained from QCM-D with the SE response and fluid-dynamics simulations, they obtained stress–strain curves for nanoscale thin films and calculated the Young’s modulus and Poisson's ratio (about 0.43). The method was further applied to fatigue testing, temperature changes, and exposure to different solvents, and was established as a comprehensive characterization protocol for ultrathin hydrogels [154] (Figure 11d).

The examples reviewed in this section highlight that the role of optical integration with QCM-based measurements is not simply to add a second readout, but to clarify the physical origin of mass and viscoelastic responses at hydrated and mechanically complex interfaces. QCM-based measurements are highly sensitive to coupled mass and mechanical changes, whereas optical readouts provide complementary information on dry mass, optical thickness, fluorescence distribution, molecular fingerprints, and spatial organization. By comparing these responses, optical–acoustic platforms can help distinguish adsorption, hydration, conformational rearrangement, lateral heterogeneity, and changes in film mechanics. Future platforms should therefore place greater emphasis on controlled measurement geometries, appropriate calibration, and coupled optical–acoustic analysis so that multimodal responses can be translated into physically meaningful information on interfacial hydration, organization, and nanoscale mechanics.

#### 4.3 Integration of optical methods with SPM

**4.3.1 TERS–SPM systems.** TERS–SPM systems link local morphological information with molecular vibrational information on the same interface, enabling nanoscale analysis of molecular chemical state, local composition, orientation, and adsorption mode [108,155–160]. Their major advantage is that they can directly visualize interfacial structures and phase-separation behavior in microscopic regions that are difficult to capture with conventional spatially averaged spectroscopy. In recent years, their application has expanded to diverse systems, including electrode interfaces [155–156] and biological membranes [158–159].

As a specific example, Mrđenović and co-workers analyzed the membrane of human pancreatic cancer cells by bottom-illumination TERS–AFM. By combining a silver-coated probe, PeakForce tapping mode, and an extremely low-power laser (160–220 μW), they minimized damage to the biological sample and visualized chemically heterogeneous structures on the membrane surface without labeling and with a spatial resolution of about 2.5 nm. As a result, they revealed spatial separation of nanodomains rich in phenylalanine, histidine, and phosphatidylcholine, as well as colocalization of cholesterol and proteins [158]. Fiocco and co-workers further applied top-illumination TERS–EC-STM to the reduction process of a (4-nitrobenzyl)mercaptan (4-NBM) monolayer in alkaline aqueous solution. By combining a partially insulated gold tip, an extremely low-power laser, and bipotentiostat control, and synchronizing a potential sweep of 50 mV·s^−1^ with rapid acquisition of Raman spectra every 0.6–1.0 s, they tracked in real time the decrease in the nitro-group band intensity and the appearance of the azo-bond band at 1452 cm^−1^, thereby demonstrating the dimerization reaction pathway [156].

In practice, however, the key challenge for TERS–SPM measurements in liquid or under operando conditions is how to avoid physical perturbation by tip contact and the occurrence of side reactions caused by laser irradiation [156–157]. Accordingly, each study adopted concrete measurement strategies suited to the target system. For example, in the cell-membrane analysis mentioned above, ex situ analysis after chemical fixation and drying was chosen in order to preserve the interfacial state by avoiding physical perturbation from the probe, with priority given to achieving the highest spatial resolution [158]. In this case, the strategy is better described as correlative sequential analysis on identical substrates rather than strictly simultaneous multimodal acquisition. Its validity depends on preserving the interfacial state through chemical fixation and on ensuring that the spectroscopic and microscopic measurements probe the same substrate region under reproducible conditions. In electrochemical operando measurements, photoinduced reactions can overlap with the intrinsic electrochemical process, but in the 4-NBM monolayer system this was suppressed successfully by combining an extremely low-power laser (about 160 μW) with constant raster scanning of the tip, allowing for reliable dynamic measurements [156].

The main value of TERS–SPM systems lies in correlating nanoscale morphology with local molecular vibrational fingerprints at the same interface, enabling analysis of chemical states, molecular orientation, adsorption modes, and nanodomain heterogeneity beyond spatially averaged spectroscopy. However, the measurement process itself can perturb the interfacial state through tip-induced disturbance, laser-induced damage or side reactions, instability under liquid or operando conditions, and structural changes during ex-situ fixation or drying. Future platforms should therefore emphasize low-power laser operation, controlled tip–sample interaction, suitable measurement protocols, and rigorous spatial registration between microscopic and spectroscopic information.

**4.3.2 SPR–AFM systems.** SPR–AFM systems combine the local topographical and force information of AFM with the effective film thickness and molecular binding kinetics of adsorbed layers obtained from plasmon resonance, thereby targeting interfacial phenomena in which macroscopic binding behavior and microscopic structural changes are intertwined [161–165].

**Figure 12:**
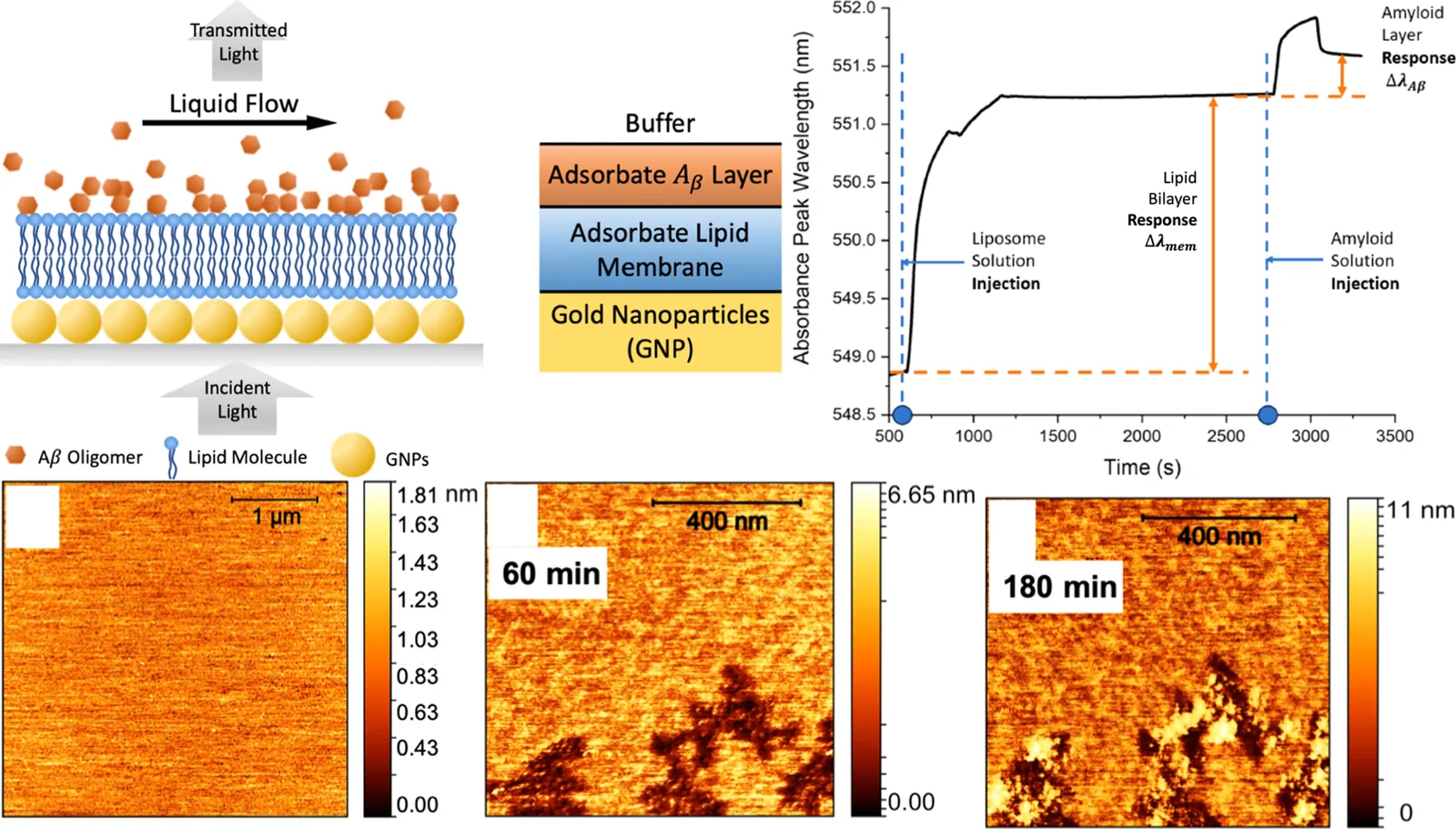
LSPR–AFM platform used for Aβ adsorption analysis. Figure 12 was adapted from [164] (© 2024 N. Mei et al., published by IOP Publishing, distributed under the terms of the Creative Commons Attribution 4.0 International License, https://creativecommons.org/licenses/by/4.0/).

Mei and co-workers combined LSPR with AFM in liquid to analyze the interaction of amyloid-β (Aβ) with artificial lipid membranes designed to mimic different stages of Alzheimer’s disease progression, as well as the protective effect of melatonin. LSPR quantified that the Aβ adsorption layer was thickest in the early disease model and that addition of 400 μM melatonin reduced the adsorption amount by about 40%, while AFM imaging in liquid visually confirmed that this macroscopic adsorption behavior originated from physical defects in the lipid membrane (pore formation) and growth of Aβ clusters [164] (Figure 12). Komorek and co-workers also evaluated BSA adsorption on gold surfaces by multiparametric SPR and AFM, and visualized pH-dependent changes in BSA molecular shape, such as elongated forms at pH 4.0 and triangular forms at pH 6.0, using contact-mode AFM with a tip radius of 20 nm. By integrating these microscopic shape data with the macroscopic molecular orientations, such as flat-on configurations, calculated from the adsorption mass measured by multiparametric SPR together with a random sequential adsorption model, they demonstrated from both perspectives the structural changes of BSA upon adsorption [161].

However, integrating these macroscopic optical responses with microscopic morphological observations requires careful attention to differences in spatial scale and to constraints associated with moving between measurement environments [164–165]. For example, in systems involving heterogeneous protein aggregates such as Aβ, the LSPR signal reflects the average refractive-index change over the entire sensor surface, forcing model-dependent interpretation as a spatially averaged “uniform effective film thickness”. For soft biological samples such as extracellular vesicles, it is often difficult to perform fully synchronized and stable measurements in liquid. In such cases, SPR imaging (SPRi) is conducted first, after which the sample is chemically fixed and transferred for AFM observation in air [165]. During this process, however, dehydration and deformation caused by the physical pressure of the probe are unavoidable.

SPR–AFM systems are particularly useful for testing whether averaged plasmonic responses arise from specific nanoscale morphological changes at the interface. In heterogeneous biological layers, changes in effective film thickness, refractive index, adsorption mass, or binding kinetics measured by SPR/LSPR/SPRi may originate from localized defects, aggregates, molecular-shape changes, or surface deformation that cannot be resolved by the optical response alone. A key requirement for future SPR–AFM platforms is therefore to preserve the native hydrated interface while matching optical and AFM observations as closely as possible in space and time, thereby reducing model-dependent interpretation and artifacts from fixation, drying, air exposure, or tip-induced deformation.

**4.3.3 Local chemical-state and morphological analysis using IR and AFM.** IR–AFM multimodal measurements complement the local topographic and nanomechanical information from AFM with molecular vibrational spectra obtained by IR spectroscopy based on photothermal expansion or near-field optical scattering [166–168]. Their major advantage is that they overcome the diffraction limit of conventional bulk IR spectroscopy and allow for direct label-free identification of the chemical state and secondary structure of single protein aggregates and cell-membrane domains on the nanoscale.

Wang and co-workers developed liquid-phase peak force infrared microscopy (LiPFIR), which integrates total internal reflection (TIR) illumination with PeakForce tapping mode. By synchronizing irradiation of a wavelength-tunable pulsed laser from below through a germanium prism only to the instant when the probe briefly contacts the sample, they eliminated lateral shear forces. This control enabled imaging with about 10 nm spatial resolution while avoiding physical damage to extremely soft biological samples such as swollen yeast cell walls and BSA fibrils; the authors also succeeded in mapping in situ the extent and local distribution of the copper-catalyzed azide–alkyne cycloaddition, a prototypical click reaction, on polymer surfaces in aqueous solution [167].

In practice, however, liquid-phase infrared measurements face major challenges in signal-to-noise ratio because of strong infrared absorption by water and the lowered Q-factor of the cantilever caused by fluid resistance [166–167]. Accordingly, each study has adopted concrete strategies suited to the target system to reduce these effects. For example, a method has been demonstrated in which live cells are measured through an ultrathin SiC/SiN membrane to physically exclude the influence of water, although local membrane-deformation artifacts caused by indentation of the probe still remain [168]. The TIR illumination mentioned above is also an effective strategy because it confines the influence of water to the near-field region, although it is subject to the physical constraint of the evanescent-field penetration depth [167]. Furthermore, in protein systems such as peptide aggregates, the solvent has been replaced with D_2_O so that the key amide-I band can be separated from the water absorption band and secondary structure can be identified [166].

The main value of IR–AFM multimodal measurements lies in correlating nanoscale topographic and nanomechanical information with local vibrational spectra, chemical states, and protein secondary structures. However, in liquid-phase measurements, optical absorption by water, cantilever damping, probe-induced deformation, and deviations from native conditions can compromise sensitivity or perturb the interfacial state. Across these optical–SPM systems, correlating local optical or spectroscopic information with nanoscale morphological and mechanical information requires not only spatial registration, but also careful consideration of how the measurement process itself affects the interfacial state. Future platforms should therefore be designed to acquire local chemical, structural, and mechanical information under native or operando conditions while preserving the native interfacial state as much as possible.

#### 4.4 Other integrated multimodal measurements: AFM–QCM-D systems

Although measurement architectures integrating AFM and QCM do not fall directly into the three major integrated categories discussed in Sections 4.1–4.3, they provide an example of other multimodal measurements in which adsorption mass and viscoelasticity at a solid–liquid interface obtained by QCM are linked with surface topography and coverage obtained by AFM, thereby enabling analysis of the relationship between interfacial coverage and wetting or fluid response [169]. In particular, they are used to analyze how polymer-adsorption dynamics at the molecular level affect drainage and rupture of hydrated thin films during bubble approach and the dynamic formation of a three-phase contact line.

**Figure 13:**
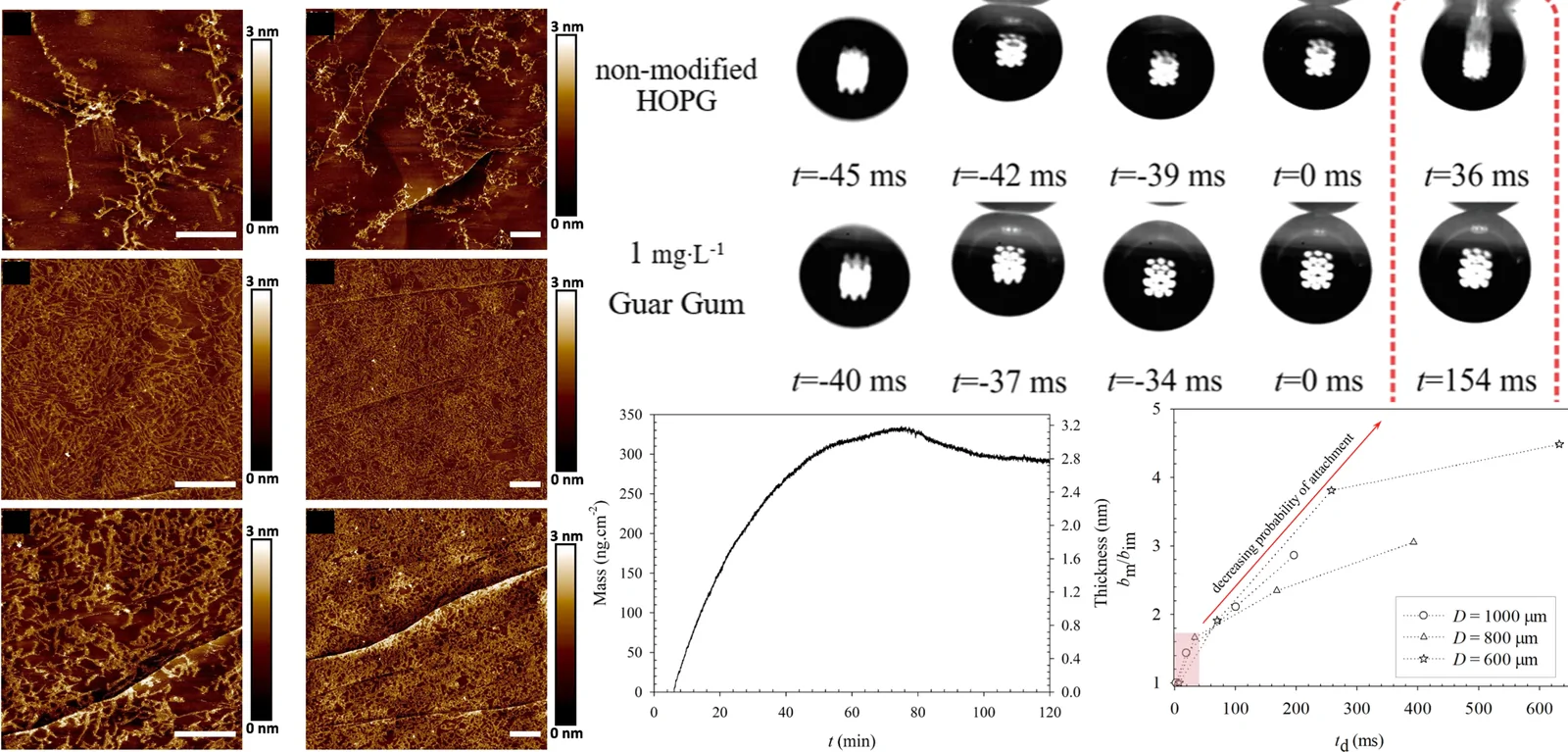
AFM–QCM-D platform used for evaluation of polymer adsorption. Figure 13 was adapted from [169] (© 2024 P. Pawliszak et al., published by Elsevier, distributed under the terms of the Creative Commons Attribution 4.0 International License, https://creativecommons.org/licenses/by/4.0/).

This complementarity was demonstrated concretely in the study by Pawliszak and co-workers. They evaluated adsorption of guar gum (GG) on hydrophobic graphite by QCM-D and AFM and showed that GG forms a concentration-dependent interconnected network with nanometer-scale thickness, changing the surface coverage from 7% to about 60%. By combining these measurements with single-bubble collision experiments recorded using a high-speed camera, nanoscale surface states were directly correlated with macroscopic fluid behavior. They found that even incomplete surface coverage altered the hydrodynamic boundary condition at the solid–liquid interface from slip to partial-slip or no-slip. This change prolonged the thin-film drainage time by up to an order of magnitude, modified the bounce amplitude, and markedly reduced the receding contact angle. At concentrations of 25 mg·L^−1^ or higher, bubble attachment was completely suppressed [169] (Figure 13).

At the same time, this method has its own technical constraints. A spatial-averaging gap can arise between the local nanoscale structures observed by AFM (1–2 μm) and the macroscopic wettability sensed by actual bubbles (diameter 400–1000 μm). In addition, estimation of adsorption mass and film thickness may depend on the assumptions of the Voigt viscoelastic model. Differences in conditions between static environments (AFM and bubble experiments) and continuous-flow environments (QCM-D) can also lead to mismatches in adsorption amount and arrival time, and these factors remain important considerations in quantitative interpretation [169].

To address the multidimensional nature of solid–liquid interfaces, researchers have strategically combined different transduction principles to bridge the gaps in observable physical quantities. A comprehensive summary of representative multimodal characterization studies discussed throughout this section is provided in Table 2. This table highlights how the integration of disparate techniques, such as the coupling of mechanical, electrochemical, and optical probes, enables the simultaneous or sequential quantification of mass, charge, and molecular conformation. These synergetic approaches provide a far more holistic view of interfacial molecular states than any single-mode measurement, as evidenced by the specific research examples listed.

**Table 2:** Representative multimodal measurement platforms at solid–liquid interfaces, together with their target physical quantities, target interfacial phenomena, spatial scales, and temporal scales.

Technique	Target physical quantities	Target interfacial phenomena	Spatial scale	Temporal scale	Ref

SPR–EC	local mass, charge	interfacial dynamics associated with adsorption, reaction, recognition, and secretion	interface: angstroms to nanometers; probing depth: tens to hundreds of nanometers; observation area: square micrometers to square millimeters, ensemble-averaged	milliseconds to seconds	[117–124]
SERS–EC	charge, molecular vibrations, orientation	intermediate transformation, molecular reorientation, phase transition, and hydration change	interface: angstroms to nanometers; observation area: square micrometers, ensemble-averaged	seconds to minutes	[21–22,125–127]
IR–EC	molecular vibrations, orientation, intermolecular interactions	hydration structure changes, biomembrane structural change (drug interaction)	interface: angstroms to nanometers; observation area: square millimeters to square centimeters, ensemble-averaged	seconds to minutes	[128–130]
VSFG–EC	local electric field/surface charge, molecular vibrations, orientation	intermediate dynamics, local electric-field response, and monolayer change	interface: angstroms to a few nanometers; observation area: square micrometers, ensemble-averaged	seconds to minutes	[131–134]
EC–time-resolved spectroscopy (ultrafast optical trigger)	photocurrent, molecular vibrations, nonlinear polarization	exciton energy transfer, charge transfer/separation, and structural relaxation	interface: angstroms to nanometers; observation area: square micrometers to square millimeters, ensemble-averaged	femtoseconds to milliseconds (partly seconds)	[135,137,139]
EC–time-resolved spectroscopy (operando potential trigger)	molecular vibrations, local electric field, faradaic charge	molecular structural changes, intermediate transformation, and electrografting	interface: angstroms to nanometers; observation area: square micrometers to square millimeters, ensemble-averaged	sub-second to tens of seconds	[136,138]
PEM	local charge/current, concentration	ion enrichment, molecular adsorption/desorption, release dynamics, and thin-film formation	pores/electrical double layer: 1–10 nm; single particles: tens to hundreds of nanometers; observation area: tens to hundreds of micrometers	milliseconds to seconds	[140]
SPR**–**QCM	mass (dry/wet), film thickness, viscoelasticity	specific/non-specific molecular adsorption and structural change	observation area: square millimeters to square centimeters, ensemble-averaged; probing depth: tens to hundreds of nanometers	a few seconds to tens of seconds	[12,20,141–147]
SERS–QCM	mass, molecular vibrations, viscoelasticity	specific molecular adsorption and structural change	observation area: square micrometers (local) to square centimeters (ensemble-averaged); probing depth: tens to hundreds of nanometers	a few seconds to tens of seconds	[148–151]
OWLS–QCM	mass (dry/wet), surface grafting density	hydration and conformational change of surface-bound polymers	observation area: square millimeters to square centimeters, ensemble-averaged; probing depth: hundreds of nanometers	several minutes	[152]
MEF–QCM	mass (surface concentration), adsorption sites (spatial distribution)	specific molecular adsorption	observation area: 1.5 μm^2^ (local) to 1.4 cm^2^ (ensemble-averaged); probing depth: tens to hundreds of nanometers	a few seconds to tens of seconds	[153]
SE–QCM	mass, optical film thickness (swelling ratio), viscoelasticity (Poisson’s ratio)	hydration, swelling, and mechanical response of ultrathin hydrogels	observation area: square millimeters to square centimeters, ensemble-averaged; probing depth: hundreds of nanometers (limit: 1 μm)	seconds to minutes	[154]
TERS–SPM	local morphology, mechanical response, molecular vibrations	nanoscale structural heterogeneity of molecular layers	lateral resolution: a few to tens of nanometers; probing depth: a few to 20 nanometers; mapping area: square micrometer scale	point: a few seconds; line: tens of seconds; map: ≤10 min	[108,155–160]
SPR–AFM	mass, local morphology, mechanical response	dynamics of interfacial molecular adsorption and morphological transformation	lateral resolution: ≤10 nm; probing depth: 20–250 nm; mapping area: square micrometer scale	SPR: real-time; map: measurement-time-dependent	[161–165]
IR–AFM	local morphology, mechanical response, molecular vibrations	dynamics of local interfacial chemistry and structural transformation	lateral resolution: ≤20 nm; probing depth: a few nanometers to several micrometers (measurement-principle-dependent); mapping area: square micrometer scale	point: several to tens of minutes; map: measurement-condition-dependent	[166–168]
AFM–QCM	mass/viscoelasticity, local structure	adsorption coverage and bubble collision/attachment response	lateral resolution: ≤2 nm; probing depth: 1–3 nm; observation area: square micrometer scale	milliseconds to seconds	[169]

### 5 Future perspectives

As discussed in Section 4, multimodal approaches that combine optics with electrochemistry, quartz crystal microbalance measurements, or scanning probe microscopy have provided a common framework for understanding solid–liquid interfaces through correlations among multiple physical quantities. Despite these important advances, current measurement technologies still face three fundamental limitations. In the following, we discuss each of these limitations and outline possible strategies to overcome them.

#### 5.1 Toward higher-order multimodal integration of interfacial state variables

Most current multimodal measurements are still limited to the integration of only two classes of physical quantities. This “two-quantity integration” has greatly deepened our understanding, but the reality of solid–liquid interfaces is more complex. Physical quantities such as charge, hydration, molecular orientation, and viscoelasticity do not change independently; they are modulated simultaneously as interlinked and non-separable state variables. For example, during protein adsorption at a solid–liquid interface, the increase in adsorbed mass (acoustic response), reorganization of the hydration layer (observed as the difference from the optical response), conformational change (vibrational spectroscopy), and change in viscoelasticity of the adsorbed layer (QCM dissipation) proceed simultaneously. At present, however, there is no technique that can extract all four quantities completely simultaneously and independently from the same probe volume.

A first direction for solving this problem is full integration of SERS, QCM-D, and electrochemistry on a single sensor chip. In recent years, integrated sensors have begun to be developed in which plasmonic nanostructures are formed directly on QCM chips, allowing for seamless realization of mass/viscoelastic measurements and Raman scattering on the same chip [148–150]. If this development is extended to a triple multimodal measurement that acquires vibrational spectroscopy, mechanical response, and electrochemical response simultaneously, it will, for the first time, become possible to capture on the same spatiotemporal scale the correlation among changes in the chemical state of adsorbed molecules, charge rearrangement at the interface, and the viscoelastic changes of the membrane induced by them. A second direction is the development of multiphysical probes that integrate TERS with electrochemical current measurements [156]. By acquiring nanoscale chemical state, mechanical response, and charge transfer in the same local space, this approach could answer simultaneously the three questions of where, what, and how things change. As a third direction, mathematical approaches that probabilistically separate independent components from averaged responses in which multiple physical quantities are superposed, by combining statistical deconvolution with machine learning, are also promising. However, since soft-modeling approaches such as multivariate curve resolution inherently suffer from rotational and intensity ambiguities, mathematical decomposition alone cannot uniquely recover true independent components. Therefore, introducing strict physical constraints or integrating independent measurements is indispensable to appropriately bound the solution. If realized, under these rigorous conditions, these approaches would provide the first true experimental breakthrough against the fundamental difficulty of the “non-separability” of state variables at solid–liquid interfaces.

#### 5.2 Bridging multimodal experiments and theoretical models

The data obtained from multimodal measurements are large and multidimensional, and their interpretation requires close coupling not only with experimental analysis but also with computational chemistry. At present, DFT calculations and MD simulations mainly play supporting roles, such as assigning vibrational bands and estimating interfacial electric fields [128–129,133–134,157]. However, a fundamental gap still remains between experiment and computation. This gap is not merely numerical. For example, QCM-D measures “wet mass”, which includes hydration water, whereas SPR reflects “dry mass”, corresponding mainly to the molecular framework. More broadly, experiments probe open, fluctuating systems and yield ensemble-averaged responses, whereas most computational models rely on closed systems with periodic boundary conditions and often describe molecular-scale trajectories under idealized conditions.

#### 5.3 Application-oriented design principles for biointerfaces, organic devices, and soft materials

If the technical advances outlined above are realized, multimodal solid–liquid interface characterization will provide new design principles for biointerfaces, delivery systems, soft materials, and organic devices. In biosensing and liquid biopsy, treating the adsorption of proteins and extracellular vesicles (EVs) as multidimensional interfacial information, including molecular orientation, hydration, viscoelasticity, and binding mass, will provide a basis for next-generation sensor platforms capable of discriminating molecular structure, functional state, and aggregation state beyond the capability of conventional mass-detection-based sensors. Early examples can already be seen in SPR–QCM-D measurements targeting tetraspanins (CD9, CD63, and CD81) in EVs derived from lung cancer cells [145] and in LSPR–AFM measurements that elucidated the membrane-disruption mechanism of amyloid-β [164]. If absolute concentrations can be quantified under complex reaction conditions and short-lived intermediates can be tracked, such approaches could substantially improve the sensitivity and specificity of biomarker detection and support the development of point-of-care diagnostics.

In drug delivery, membrane science, and biomaterials, multimodal platforms capable of quantitatively tracking liposome and lipid nanoparticle fusion with cell membranes, together with cargo-release dynamics, through correlated mass, optical, and electrochemical signals will directly support the rational design of delivery carriers. Likewise, quantifying the relationships among surface hydration, viscoelasticity, and nonspecific protein adsorption will accelerate the development of biocompatible materials and antifouling coatings through the rational design of hydration networks. Beyond these biorelated systems, similar multimodal strategies may also transform the analysis of organic thin-film devices, including organic field-effect transistors and organic electroluminescent devices, by revealing how potential-dependent conformational changes in organic molecular layers are coupled to charge-transport properties. Solid–liquid interface science is thus evolving from the study of local boundary phenomena into an integrated discipline that provides molecular-level design principles for biointerfaces, soft materials, and organic devices.

## Conclusion

Multimodal characterization has fundamentally shifted the description of solid–liquid interfaces from a uniform, averaged picture toward one based on locally heterogeneous and dynamically correlated structures. By revealing that interfacial phenomena are inherently coupled, multistep processes, these integrated methods have proven essential for capturing the complex realities of interfacial systems. However, moving forward, the true impact of this field will depend on addressing several unresolved challenges. First, the relevant interfacial state variables are physically coupled and not easily separable, making it difficult to infer causal relationships among charge, structure, and hydration from experimental data alone. Second, nanoscale spatial resolution and ultrafast temporal resolution remain difficult to achieve simultaneously, and no current method can fully reveal both where interfacial reactions occur and how they evolve in real time. Third, quantitative integration between experiment and theory is still limited by fundamental differences in physical definitions and boundary conditions, including the distinction between the hydration-inclusive wet mass measured by QCM-D and the dry mass detected by SPR, as well as the mismatch between open experimental systems and closed computational models.

To address these challenges, three future directions appear particularly important. The first is the realization of true simultaneous acquisition of multiple physical quantities through deeper multimodal integration, for example by combining SERS, QCM-D, and EC on a single platform or by developing multiphysical TERS probes. The second is to overcome the current trade-off in spatiotemporal resolution through pulse-laser-synchronized TERS and super-resolution tracking approaches. The third is to establish a framework for quantitative integration of experiment and theory through inverse modeling combined with molecular dynamics simulations and deep learning. Progress along these directions should accelerate the emergence of a new measurement paradigm capable of linking temporal and spatial scales while simultaneously capturing multiple interfacial observables in real time.

We hope that the conceptual framework and design principles summarized in this review will support further advances in molecular science at solid–liquid interfaces and contribute to the rational design of biosensing platforms, delivery systems, biocompatible materials, and organic devices.

## Data Availability

Data sharing is not applicable as no new data was generated or analyzed in this work.

## References

[1] Yamakata A, Osawa M (2008). J Phys Chem C.

[2] Greco A, Imoto S, Backus E H G, Nagata Y, Hunger J, Bonn M (2025). Science.

[3] Yamakata A, Soeta E, Ishiyama T, Osawa M, Morita A (2013). J Am Chem Soc.

[4] Maitra A, Pennathur A K, Chiang P-H, Dawlaty J M (2024). ACS Appl Energy Mater.

[5] Wertz C F, Santore M M (2002). Langmuir.

[6] Wertz C F, Santore M M (2001). Langmuir.

[7] Son Y J, Han J W, Kang H, Seong S, Han S, Maeda S, Chikami S, Hayashi T, Hara M, Noh J (2023). Int J Mol Sci.

[8] Reimhult E, Larsson C, Kasemo B, Höök F (2004). Anal Chem (Washington, DC, U S).

[9] Chang A L, McKeague M, Liang J C, Smolke C D (2014). Anal Chem (Washington, DC, U S).

[10] Butt M A (2025). Biosensors.

[11] Bonet N F, Cava D G, Vélez M (2022). Front Mol Biosci.

[12] Komorek P, Martin E, Jachimska B (2021). Int J Mol Sci.

[13] Glassford S E, Byrne B, Kazarian S G (2013). Biochim Biophys Acta, Proteins Proteomics.

[14] Guo W, Lu T, Crisci R, Nagao S, Wei T, Chen Z (2023). Chem Sci.

[15] Peng J, Guo J, Ma R, Jiang Y (2022). Surf Sci Rep.

[16] Rahimi E, PalaciosCorella M, Mol A, Pané S, Puigmartí-Luis J (2025). Adv Mater (Weinheim, Ger).

[17] Kumar N, Zheng L-Q, Pollard A J, Wain A J, Zenobi R (2026). Chem Rev.

[18] Nakatsuka N, Yang K-A, Abendroth J M, Cheung K M, Xu X, Yang H, Zhao C, Zhu B, Rim Y S, Yang Y (2018). Science.

[19] Lim K, Fenk B, Popovic J, Maier J (2021). ACS Appl Mater Interfaces.

[20] Komorek P, Wałek M, Jachimska B (2020). Bioelectrochemistry.

[21] Tran C T, Nguyen L B T, Tan E X, Preiser P R, Phang I Y, Ling X Y (2026). J Am Chem Soc.

[22] Wicaksono W P, Dang H, Lee S, Choo J (2024). Appl Surf Sci.

[23] Singh S N, Yadav S, Shire S J, Kalonia D S (2014). Pharm Res.

[24] Laue T M, Shire S J (2020). J Pharm Sci.

[25] Wu J (2022). Chem Rev.

[26] Adamczyk Z (2003). Adv Colloid Interface Sci.

[27] Agmo Hernández V (2023). ChemTexts.

[28] Eom N, Parsons D F, Craig V S J (2017). J Phys Chem B.

[29] Grasso D, Subramaniam K, Butkus M, Strevett K, Bergendahl J (2002). Rev Environ Sci Technol.

[30] Tabor R F, Wu C, Grieser F, Dagastine R R, Chan D Y C (2013). J Phys Chem Lett.

[31] Kanth J M P, Vemparala S, Anishetty R (2010). Phys Rev E.

[32] Zhang Y, Furyk S, Bergbreiter D E, Cremer P S (2005). J Am Chem Soc.

[33] Blesa M A, Weisz A D, Morando P J, Salfity J A, Magaz G E, Regazzoni A E (2000). Coord Chem Rev.

[34] Reddish M J, Vaughn M B, Fu R, Dyer R B (2016). Biochemistry.

[35] Maus H, Hinze G, Hammerschmidt S J, Schirmeister T, Basché T (2023). ChemistryEurope.

[36] Dogan J, Schmidt T, Mu X, Engström Å, Jemth P (2012). J Biol Chem.

[37] Roach P, Farrar D, Perry C C (2005). J Am Chem Soc.

[38] Larsericsdotter H, Oscarsson S, Buijs J (2004). J Colloid Interface Sci.

[39] Du X, Li Y, Xia Y-L, Ai S-M, Liang J, Sang P, Ji X-L, Liu S-Q (2016). Int J Mol Sci.

[40] Gobbi M, Galanti A, Stoeckel M-A, Zyska B, Bonacchi S, Hecht S, Samorì P (2020). Nat Commun.

[41] Boldt F-M, Baltes N, Borgwarth K, Heinze J (2005). Surf Sci.

[42] Camacho C J, Kimura S R, DeLisi C, Vajda S (2000). Biophys J.

[43] Khalid-Salako F, Kurt H, Yüce M (2026). ACS Omega.

[44] Davantès A, Nigen M, Sanchez C, Renard D (2023). Colloids Interfaces.

[45] Arteta M Y, Berti D, Montis C, Campbell R A, Eriksson C, Clifton L A, Skoda M W A, Soltwedel O, Koutsioubas A, Baglioni P (2015). Soft Matter.

[46] Kurkina T, Vlandas A, Ahmad A, Kern K, Balasubramanian K (2011). Angew Chem, Int Ed.

[47] Sivakumarasamy R, Hartkamp R, Siboulet B, Dufrêche J-F, Nishiguchi K, Fujiwara A, Clément N (2018). Nat Mater.

[48] Pham C D, Nguyen T T H, Zhang J, Jamali S, Nguyen N-T, Nguyen T-K (2026). ACS Appl Nano Mater.

[49] Dhopatkar N, Defante A P, Dhinojwala A (2016). Sci Adv.

[50] Wu G, Fang J, Shrawankar K, Guo W, Nagao S, Chen X, Wu Y, Wei T, Chen Z (2026). Anal Chem (Washington, DC, U S).

[51] Dunér G, Thormann E, Dėdinaitė A (2013). J Colloid Interface Sci.

[52] Jordan J L, Fernandez E J (2008). Biotechnol Bioeng.

[53] Doudevski I, Schwartz D K (2001). J Am Chem Soc.

[54] Dziubak D, Strzelak K, Sek S (2020). Membranes.

[55] Kujawa P, Moraille P, Sanchez J, Badia A, Winnik F M (2005). J Am Chem Soc.

[56] Gurdak E, Dupont-Gillain C C, Booth J, Roberts C J, Rouxhet P G (2005). Langmuir.

[57] Sarcina L, Torsi L, Picca R A, Manoli K, Macchia E (2020). Sensors.

[58] Politano G G, Versace C (2023). Spectrosc J.

[59] Mora M F, Giacomelli C E, Garcia C D (2009). Anal Chem (Washington, DC, U S).

[60] Höök F, Vörös J, Rodahl M, Kurrat R, Böni P, Ramsden J J, Textor M, Spencer N D, Tengvall P, Gold J (2002). Colloids Surf, B.

[61] Häbich A, Qiao G G, Ducker W (2010). Langmuir.

[62] Roach P, Farrar D, Perry C C (2006). J Am Chem Soc.

[63] Lyu K, Chen H, Gao J, Jin J, Shi H, Schwartz D K, Wang D (2022). Biomacromolecules.

[64] Pandey S, Gautam D, Chen J (2024). J Phys Chem B.

[65] Bouzigues C I, Tabeling P, Bocquet L (2008). Phys Rev Lett.

[66] Wang T, Jiang Y, Feng H, Liu L, Deng Q, Liu D, Wang C (2025). Catalysts.

[67] Brulé T, Bouhelier A, Dereux A, Finot E (2016). ACS Sens.

[68] Niu T, Yuan Y, Yao J, Lu F, Gu R (2011). Sci China: Chem.

[69] Liu C, Lei F, Gong M, Zhou X, Zhao X, Li Z, Zhang C, Man B, Yu J (2024). Energy Environ Mater.

[70] Miranda P B, Shen Y R (1999). J Phys Chem B.

[71] Tian C S, Shen Y R (2009). Proc Natl Acad Sci U S A.

[72] Rehl B, Li Z, Gibbs J M (2018). Langmuir.

[73] Eisenthal K B (1996). Chem Rev.

[74] Konek C T, Musorrafiti M J, Al-Abadleh H A, Bertin P A, Nguyen S T, Geiger F M (2004). J Am Chem Soc.

[75] Fitts J P, Shang X, Flynn G W, Heinz T F, Eisenthal K B (2005). J Phys Chem B.

[76] Escorihuela J, González-Martínez M Á, López-Paz J L, Puchades R, Maquieira Á, Gimenez-Romero D (2015). Chem Rev.

[77] Cross G H, Reeves A A, Brand S, Popplewell J F, Peel L L, Swann M J, Freeman N J (2003). Biosens Bioelectron.

[78] Zheng Y, Ma L, Wang Y, Huang J, Ye J, Yang X (2025). Anal Chem (Washington, DC, U S).

[79] Yanase Y, Hiragun T, Ishii K, Kawaguchi T, Yanase T, Kawai M, Sakamoto K, Hide M (2014). Sensors.

[80] Plikusiene I, Maciulis V, Ramanavicius A, Ramanaviciene A (2022). Polymers (Basel, Switz).

[81] Bieberle-Hütter A, Bronneberg A C, George K, van de Sanden M C M (2021). J Phys D: Appl Phys.

[82] Colson L, Kwon Y, Nam S, Bhandari A, Maya N M, Lu Y, Cho Y (2023). Sensors.

[83] Lazanas A C, Prodromidis M I (2023). ACS Meas Sci Au.

[84] Magar H S, Abbas M N, Ali M B, Ahmed M A (2020). J Solid State Electrochem.

[85] Syu Y-C, Hsu W-E, Lin C-T (2018). ECS J Solid State Sci Technol.

[86] Minamiki T, Minami T, Kurita R, Niwa O, Wakida S-i, Fukuda K, Kumaki D, Tokito S (2014). Appl Phys Lett.

[87] Wang S-L, Hsieh C-Y, Wu C-R, Chen J-C, Wang Y-L (2021). Biomicrofluidics.

[88] Zhang X, Han C, Xu W (2023). Chem Biomed Imaging.

[89] Roberts W S, Davis F, Collyer S D, Higson S P J (2011). Analyst.

[90] Janićijević Ž, Nguyen-Le T-A, Baraban L (2023). Next Nanotechnol.

[91] Putnam S T, Santiago-Carboney A, Qian P, Rodríguez-López J (2025). Anal Chem (Washington, DC, U S).

[92] Lubarsky G V, Davidson M R, Bradley R H (2007). Biosens Bioelectron.

[93] Drew M E, Chworos A, Oroudjev E, Hansma H, Yamakoshi Y (2010). Langmuir.

[94] Andriotis O G, Elsayad K, Smart D E, Nalbach M, Davies D E, Thurner P J (2019). Biomed Opt Express.

[95] Nishide G, Lim K, Kobayashi A, Qiu Y, Hazawa M, Ando T, Okada Y, Wong R W (2025). Nucleic Acids Res.

[96] Ruan Y, Kao K, Lefebvre S, Marchesi A, Corringer P-J, Hite R K, Scheuring S (2018). Proc Natl Acad Sci U S A.

[97] Fukuma T, Garcia R (2018). ACS Nano.

[98] Asakawa H, Yoshioka S, Nishimura K-i, Fukuma T (2012). ACS Nano.

[99] Fukuma T, Ueda Y, Yoshioka S, Asakawa H (2010). Phys Rev Lett.

[100] Araki Y, Sekine T, Chang R, Hayashi T, Onishi H (2018). RSC Adv.

[101] Hackl T, Schitter G, Mesquida P (2022). ACS Nano.

[102] Hackl T, Poik M, Schitter G (2023). IEEE Trans Instrum Meas.

[103] Zheng L-Q, Servalli M, Schlüter A D, Zenobi R (2019). Chem Sci.

[104] Kurouski D, Deckert-Gaudig T, Deckert V, Lednev I K (2012). J Am Chem Soc.

[105] El-Khoury P Z (2023). J Am Chem Soc.

[106] Müller D J, Dumitru A C, Lo Giudice C, Gaub H E, Hinterdorfer P, Hummer G, De Yoreo J J, Dufrêne Y F, Alsteens D (2021). Chem Rev.

[107] Ai Q, Bonagiri L K S, Farokh Payam A, Aluru N R, Zhang Y (2025). J Phys Chem C.

[108] Martín Sabanés N, Driessen L M A, Domke K F (2016). Anal Chem (Washington, DC, U S).

[109] Cousin F, Fadda G (2020). EPJ Web Conf.

[110] Pan F, Aaron Lau K H, Messersmith P B, Lu J R, Zhao X (2020). Langmuir.

[111] Li Z, Saurabh S, Hollowell P, Kalonia C K, Waigh T A, Li P, Webster J R P, Seddon J M, Bresme F, Lu J R (2024). ACS Appl Mater Interfaces.

[112] Skoda M W A (2019). Curr Opin Colloid Interface Sci.

[113] Khassanov A, Steinrück H-G, Schmaltz T, Magerl A, Halik M (2015). Acc Chem Res.

[114] Richter A G, Kuzmenko I (2013). Langmuir.

[115] Velasco-Velez J-J, Wu C H, Pascal T A, Wan L F, Guo J, Prendergast D, Salmeron M (2014). Science.

[116] Caselli L, Nylander T, Malmsten M (2024). Adv Colloid Interface Sci.

[117] Hatami D, Hasler R, Spanu A, Dostalek J, Kleber C, Knoll W, Bonfiglio A (2025). Sci Rep.

[118] Zhao R, Yan B, Li D, Guo Z, Huang Y, Wang D, Yao X (2024). Anal Chem (Washington, DC, U S).

[119] Thakur B, Anbalagan A C, Koyande P, Sawant S N (2025). Sci Rep.

[120] Yan B, Han X, Zhao R, Guo Z, Li D, Yan Y, Wang D, Yao X (2025). Anal Chem (Washington, DC, U S).

[121] Ma S-C, Gupta R, Ondevilla N A P, Barman K, Lee L-Y, Chang H-C, Huang J-J (2023). Biomed Opt Express.

[122] Jungnickel R, Balasubramanian K (2024). Adv Sens Res.

[123] Fritz P A, Bera B, van den Berg J, Visser I, Kleijn J M, Boom R M, Schroën C G P H (2021). Langmuir.

[124] Gibson J S, Mendes P M (2021). ChemPhysChem.

[125] Zhan C, Dattila F, Rettenmaier C, Bergmann A, Kühl S, García-Muelas R, López N, Cuenya B R (2021). ACS Catal.

[126] Wu J, Zhou R, Radjenovic P M, Liu S, Wu D, Li J, Mao B, Yan J (2021). Electrochim Acta.

[127] Zhu C, Fan C, Cortés E, Xie W (2021). J Mater Chem A.

[128] Alvarez-Malmagro J, Ruano L, Cuartero-González M, Nogueira J J, Prieto-Dapena F (2025). J Phys Chem B.

[129] Pudžaitis V, Talaikis M, Sadzevičienė R, Labanauskas L, Niaura G (2022). Materials.

[130] Su Z, Chen A, Lipkowski J (2024). Langmuir.

[131] Liu X, Ma Y, Song Q, Huang H, He Y, Sun S, Wang Z (2023). J Phys Chem C.

[132] Patrow J G, Sorenson S A, Dawlaty J M (2017). J Phys Chem C.

[133] Ge A, Videla P E, Lee G L, Rudshteyn B, Song J, Kubiak C P, Batista V S, Lian T (2017). J Phys Chem C.

[134] Bhattacharyya D, Videla P E, Palasz J M, Tangen I, Meng J, Kubiak C P, Batista V S, Lian T (2022). J Am Chem Soc.

[135] Bodine M, Rozyyev V, Elam J W, Tokmakoff A, Lewis N H C (2023). J Phys Chem C.

[136] Unni B, Burgess I J (2021). Electrochim Acta.

[137] Nawrocki W J, Jones M R, Frese R N, Croce R, Friebe V M (2023). Joule.

[138] Siddiqui A-R, N’Diaye J, Martin K, Baby A, Dawlaty J, Augustyn V, Rodríguez-López J (2024). Anal Chem (Washington, DC, U S).

[139] López-Ortiz M, Bolzonello L, Bruschi M, Fresch E, Collini E, Hu C, Croce R, van Hulst N F, Gorostiza P (2024). ACS Appl Mater Interfaces.

[140] Groysman S, Chen Y, Garcia A, Martinez C, Diego-Perez K, Benavides M, Chen Y, Wan Z, Wang S, Liu R (2025). ACS Electrochem.

[141] Fernandes E, Costa R R, Machado R, Reis R L, Pashkuleva I, Lúcio M (2024). Small Sci.

[142] Spagnolo S, Sontakke K, Dubbert L, Urban M, Lednicky T, Csaki A, Wondraczek K, Fritzsche W, Hianik T (2025). Biosensors.

[143] Asai N, Matsumoto N, Yamashita I, Shimizu T, Shingubara S, Ito T (2021). Sens Bio-Sens Res.

[144] Wang P, Liu T, Liu Y, Tian J, Zhang X, Guo J, Jin Y, Xiao H, Song J (2021). Sens Actuators, B.

[145] Kowalczyk A, Gajda-Walczak A, Ruzycka-Ayoush M, Targonska A, Mosieniak G, Glogowski M, Szumera-Cieckiewicz A, Prochorec-Sobieszek M, Bamburowicz-Klimkowska M, Nowicka A M (2023). Anal Chem (Washington, DC, U S).

[146] Arrizabalaga J, Rafaniello I, Schäfer T (2025). J Membr Sci.

[147] Yoon B K, Ma G J, Park H, Ferhan A R, Cho N-J, Jackman J A (2021). Int J Biol Macromol.

[148] Bartolini C, Tozzetti M, Gellini C, Ricci M, Menichetti S, Procacci P, Caminati G (2025). Nanomaterials.

[149] Hou L, Xu X, Tian F, Xu Y (2025). Talanta.

[150] Lequeux M, Salmain M, Krafft J-M, Dostalek J, Vallée A, Loiseau A, Knoll W, Lamy de la Chapelle M, Boujday S (2025). Sens Actuators, B.

[151] Armero L, Plou J, Valera P S, Serna S, García I, Liz-Marzán L M (2024). ACS Sens.

[152] Perrino C, Lee S, Spencer N D (2024). Langmuir.

[153] Rastogi R, Beggiato M, Dogbe Foli E A, Vincent R, Dupont-Gillain C, Adam P-M, Krishnamoorthy S (2021). J Phys Chem C.

[154] Sans J, Azevedo Gonçalves I, Quintana R (2024). Small.

[155] Dinda S, Trivedi S, Roy A, Pammer F D, Fichtner M (2023). Adv Energy Mater.

[156] Fiocco A, Pavlic A A, Kanoufi F, Maisonhaute E, Noël J-M, Lucas I T (2024). Anal Chem (Washington, DC, U S).

[157] Bartolomeo G L, Zhang Y, Kumar N, Zenobi R (2021). Anal Chem (Washington, DC, U S).

[158] Mrđenović D, Ge W, Kumar N, Zenobi R (2022). Angew Chem, Int Ed.

[159] Pandey Y, Kumar N, Goubert G, Zenobi R (2021). Angew Chem, Int Ed.

[160] Proniewicz E, Olszewski T K (2022). J Med Chem.

[161] Komorek P, Rakowski K, Szota M, Lekka M, Jachimska B (2023). bioRxiv.

[162] Makou E, Bailey R G, Johnston H, Parkin J D, Hulme A N, Hähner G, Barlow P N (2019). J Biol Chem.

[163] Szczepanczyk M, Gonzalez-Martinez J F, Ruzgas T, Björklund S (2026). J Colloid Interface Sci.

[164] Mei N, Liang J, McRae D M, Leonenko Z (2024). Nanotechnology.

[165] Raizada G, Guillouzouic J, Rouleau A, Lesniewska E, Le Ferrec E, Elie-Caille C, Boireau W (2025). Biosensors.

[166] Ramer G, Ruggeri F S, Levin A, Knowles T P J, Centrone A (2018). ACS Nano.

[167] Wang H, González-Fialkowski J M, Li W, Xie Q, Yu Y, Xu X G (2021). Anal Chem (Washington, DC, U S).

[168] Veber A, Spedalieri C, Kneipp J (2025). Small.

[169] Pawliszak P, Beheshti A, Møller A, Blencowe A, Beattie D A, Krasowska M (2024). J Colloid Interface Sci.

